# The Mediator complex kinase module is necessary for fructose regulation of liver glycogen levels through induction of glucose-6-phosphatase catalytic subunit (*G6pc*)

**DOI:** 10.1016/j.molmet.2021.101227

**Published:** 2021-03-31

**Authors:** Dou Yeon Youn, Alus M. Xiaoli, Haihong Zong, Junichi Okada, Li Liu, Jacob Pessin, Jeffrey E. Pessin, Fajun Yang

**Affiliations:** 1 Department of Medicine, Albert Einstein College of Medicine, Bronx, NY, 10461, USA; 2 Department of Molecular Pharmacology, Albert Einstein College of Medicine, Bronx, NY, 10461, USA; 3 Department of Developmental and Molecular Biology, Albert Einstein College of Medicine, Bronx, NY, 10461, USA; 4 Fleischer Institute for Diabetes and Metabolism, Albert Einstein College of Medicine, Bronx, NY, 10461, USA

**Keywords:** Mediator complex, Kinase module, Fructose, *G6pc*, Liver glycogen

## Abstract

**Objective:**

Liver glycogen levels are dynamic and highly regulated by nutrient availability as the levels decrease during fasting and are restored during the feeding cycle. However, feeding in the presence of fructose in water suppresses glycogen accumulation in the liver by upregulating the expression of the glucose-6-phosphatase catalytic subunit (*G6pc*) gene, although the exact mechanism is unknown. We generated liver-specific knockout MED13 mice that lacked the transcriptional Mediator complex kinase module to examine its effect on the transcriptional activation of inducible target gene expression, such as the ChREBP- and FOXO1-dependent control of the *G6pc* gene promoter.

**Methods:**

The relative changes in liver expression of lipogenic and gluconeogenic genes as well as glycogen levels were examined in response to feeding standard low-fat laboratory chow supplemented with water or water containing sucrose or fructose in control (*Med13*^fl/fl^) and liver-specific MED13 knockout (MED13-LKO) mice.

**Results:**

Although MED13 deficiency had no significant effect on constitutive gene expression, all the dietary inducible gene transcripts were significantly reduced despite the unchanged insulin sensitivity in the MED13-LKO mice compared to that in the control mice. *G6pc* gene transcription displayed the most significant difference between the *Med13* ^fl/fl^ and MED13-LKO mice, particularly when fed fructose. Following fasting that depleted liver glycogen, feeding induced the restoration of glycogen levels except in the presence of fructose. MED13 deficiency rescued the glycogen accumulation defect in the presence of fructose. This resulted from the suppression of *G6pc* expression and thus G6PC enzymatic activity.

Among two transcriptional factors that regulate *G6pc* gene expression, FOXO1 binding to the *G6pc* promoter was not affected, whereas ChREBP binding was dramatically reduced in MED13-LKO hepatocytes. In addition, there was a marked suppression of FOXO1 and ChREBP-β transcriptional activities in MED13-LKO hepatocytes.

**Conclusions:**

Taken together, our data suggest that the kinase module of the Mediator complex is necessary for the transcriptional activation of metabolic genes such as *G6pc* and has an important role in regulating glycogen levels in the liver through altering transcription factor binding and activity at the *G6pc* promoter.

## Highlights

• *G6pc* is upregulated with fructose supplementation through the ChREBP-dependent pathway.
• Upregulation of *G6pc* suppresses glycogen synthesis in the liver.
• Knockout of the Mediator complex MED13 subunit inhibits *G6pc* gene expression.
• The Mediator complex kinase module is required for ChREBP-dependent activation of the *G6pc* gene.

## 1 Introduction

Different types of dietary carbohydrates have marked differential effects on metabolism and the development of obesity, diabetes, fatty liver, and cardiovascular diseases [[1], [2], [3]]. In particular, fructose is a more potent driver of *de novo* lipogenesis in the liver than glucose primarily through a greater induction of lipogenic gene expression through the activation of the transcription factors, sterol regulatory element-binding protein 1c (SREBP-1c) and carbohydrate response element-binding protein (ChREBP) [[4], [5], [6], [7]]. While both glucose and fructose are phosphorylated to fructose 6-phosphate (F6P) and fructose 1-phosphate (F1P), respectively, only glucokinase but not fructokinase (ketohexokinase) is highly sensitive to its end-product inhibition [[8], [9], [10]]. It was recently reported that at low doses, the major absorption and metabolism of dietary fructose occurs in the small intestines, whereas at high doses, fructose saturates intestinal metabolism resulting in excess fructose primarily going to and being metabolized by the liver [11,12]. The liver contains high levels of aldolase B that rapidly hydrolyzes F1P to dihydroacetone phosphate (DHAP) and glyceraldehyde [13]. The rapid phosphorylation of fructose results in depletion of ATP and subsequent activation of the AMP-dependent protein kinase (AMPK), nucleotide degradation, and uric acid formation [[14], [15], [16], [17]]. AMPK is an important energy sensor that suppresses the mechanistic target of rapamycin complex 1 (mTORC1), another important energy-sensing signaling node [18]. Because insulin induction of SREBP1c-mediated lipogenic gene expression depends on mTORC1, fructose induction of lipogenic gene likely results from a distinct pathway. Fructose was recently shown to induce the expression of the constitutively active ChREBP-β transcription factor and lipogenic gene expression through the nuclear translocation of ChREBP-α independent from mTORC1 [7,19]. Importantly, the catalytic subunit of glucose 6-phosphatase (*G6pc*) is a critical gluconeogenic gene and is also transactivated by ChREBP-β [20,21]. These findings suggest a pathophysiologic basis for the elevation of both *de novo* lipogenesis and increased hepatic glucose production (glycogenolysis and gluconeogenesis) that occurs in insulin-resistant states.

However, most eukaryotic transcription factors, including SREBP-1c and ChREBP-β, are unable to directly contact and activate RNA polymerase II (Pol II) required for mRNA transcription. The Mediator complex links these and the vast majority of regulated transcriptional factors and co-regulators with the basal transcriptional machinery [22,23]. The small or core mammalian Mediator complex contains 26 subunits whereas the large Mediator complex consists of the core Mediator without MED26 plus the kinase module composed of MED13, MED12, CDK8, and cyclin C (CCNC) [[24], [25], [26]]. Under normal physiologic conditions, the Mediator complex in the mouse liver undergoes a dynamic nutrient and hormonal-dependent interconversion between the large and small Mediator complex regulated through the mTORC1 pathway [27]. The relative contribution of the large and small Mediator complex to Pol II-dependent transcription has remained enigmatic and appears to result from complex interactions between combinatorial factors in a cell context-dependent manner [[28], [29], [30]]. In the present study, we used liver-specific MED13 knockout mice that resulted in the complete loss of the Mediator complex kinase module and generated the constitutive formation of the small/core Mediator. In these mice, we demonstrated that the kinase module is necessary for the transcriptional activation of *G6pc* expression and protects mice against some deleterious effects of fructose.

## 2 Materials and methods

### 2.1 Animals

All the animal experiments followed guidelines that were approved and in agreement with the Albert Einstein College of Medicine Institutional Animal Care and Use Committee (2016–0901). *Med13*^*fl*/fl^ mice were kindly provided by Dr. Eric Olson and backcross-bred with C57BL/6J from Jackson Laboratories for 8 generations. MED13 liver-specific knockout mice (MED13-LKO) were generated by cross-breeding with C57BL/6J. *Albumin*-Cre (*Alb*-Cre) mice were purchased from Jackson Laboratories. For *G6pc* overexpression in the *Med13*^fl/fl^ and MED13-LKO mice, AAV8-*Tbg-G6pc* was cloned and packaged (Vector Biolabs) for intravenous injection at 5 × 10^11^ GC/mouse. The mice had free access to food and water unless otherwise noted. All of the mice were housed in groups and under a facility equipped with a 12-h light/dark cycle.

Standard laboratory mouse chow (LabDiet #5053) that contained 25% protein, 13% fat, and 62% carbohydrates was provided for the mice. Prior to feeding experiments, the mice were trained for 3 days by removing food at 5 PM and feeding at 9 AM the next day (16-h fast overnight and 8-h feeding daytime). On the day of the experiment, the fasting group of mice was sacrificed in the morning at 11 AM (16-h fast) while the 9 AM fed group was also provided drinking water that contained 30% sucrose or fructose (w/v). The fed mice were sacrificed at 1 PM equal to 4 h of feeding. Then 30% sucrose or fructose water was prepared (w/v) and provided to the mice on the day of the experiment and not during the fasting/feeding training period.

For glucose, insulin, and pyruvate tolerance tests, mice were fasted overnight for 16 h (glucose tolerance test) or 6 h (insulin and pyruvate tolerance test) and tests were performed as previously described [31]. Blood glucose was determined by a Precision Xtra (Abbott) glucose-monitoring system, and plasma insulin levels were measured by an Ultra-Sensitive Mouse Insulin ELISA kit (Crystalchem). The respiratory exchange ratio was determined by indirect calorimetry using a Columbus Instruments Oxymax system as previously described [32].

### 2.2 Antibodies and immunoblotting

Antibodies against the following proteins were used in this study: MED13 (1:1000, Fisher PA5-35924 or 1:1000 Bethyl A301-278A), MED12 (1:1000, Abcam ab70842), CDK8 (1:1000, Abcam ab54561 or 1:1000, Bethyl A302-501A-M), CCNC (1:1000, Bethyl A301-989A), MED15 (1:1000, Bethyl A302-422A), nucleolin (1:1000, Cell Signaling 14574S), tubulin (1:1000, Cell Signaling 2144S), ChREBP (1:1000, Novus Biologicals NB400–135SS), phospho-S6 ribosomal protein (1:1000, Cell Signaling 4858P), S6 ribosomal protein (1:1000, Cell Signaling 2317S), phospho-AKT S473 (1:1000, Cell Signaling 4060), T308 (1:1000, Cell Signaling 4056), T450 (1:1000, Cell Signaling 12,178), total AKT (1:1000, Cell Signaling 9272), phospho GS (S641, 1:1000 Cell Signaling 3891), GS (1:1000 Cell Signaling 3893), phospho FOXO1 (S256, 1:1000 Cell Signaling 84,192), and FOXO1 (1:1000 Cell Signaling 2880). Total liver tissue lysates were generated using Pierce IP lysis buffer (87,787, Thermo Fisher Scientific) with freshly added 1 mM of dithiothreitol (DTT), protease inhibitors, and phosphatase inhibitors. Then, 3–8% Tris-acetate gels (Invitrogen) and nitrocellulose membranes (Invitrogen, iBlot gel transfer) were used to analyze protein samples. Western blotting signals were detected with either goat anti-rabbit or goat anti-mouse secondary antibodies (IRDye 800CW) from LI-COR. For a densitometric analysis, the relative band intensity was quantified by ImageJ and normalized to the loading control nucleolin or tubulin.

### 2.3 RNA isolation and quantitative PCR

Total RNA isolation from mouse livers and a quantitative PCR analysis were performed as previously described [33]. Briefly, an RNeasy purification kit (Qiagen) was used for RNA purification according to the manufacturer's protocol. A SuperScript VILO cDNA synthesis kit (Invitrogen) and PowerUp SYBR Green Master Mix (Applied Biosystems) were used for cDNA strand synthesis and a qPCR and StepOnePlus Real-Time PCR system (Applied Biosystems) was used for quantification. Samples were normalized to the *Ppib* gene to determine the relative mRNA levels. The primer sequences used in this study are listed in Table S1.

### 2.4 Nuclear extraction and glycerol gradient fractionation

All of the procedures were performed on ice in a 4 °C cold room. All the buffers contained a cocktail of protease inhibitors (Sigma Aldrich), phosphatase inhibitors (Sigma Aldrich), and 1 mM of DTT and were made immediately before use. Fresh mouse liver nuclear extracts were prepared as previously described with some modifications [34]. Briefly, the pooled liver extracts for each condition were loaded directly onto a glycerol gradient column generated by an auto mix gradient former (Jule) with 40% and 80% (w/v) glycerol in gradient buffer (20 mM of Hepes, 100 mM of NaCl, and 1.5 mM of MgCl_2_, pH 7.8) on top of a 200 μL cushion of 100% glycerol. Gradients containing samples or native marker protein standards that ran in parallel (Invitrogen) were centrifuged at 16,000×*g* for 16 h at 4 °C in a Beckman ultracentrifuge with SW55 Ti or VTi 65.2 rotors. Gradient fractions (50–100 μL) were collected from the top of the column and analyzed by Western blotting. Fractions taken from the protein standard column were resolved on NuPAGE 3–8% Tris-acetate native gel (Thermo Fisher Scientific) followed by silver staining and compared with the fractions from the lysate samples.

### 2.5 Chromatin immunoprecipitation (ChIP) assay

ChIP was performed as previously described [35]. Briefly, cross-linked mouse liver nuclear samples were sonicated in TE buffer containing protease inhibitor (Sigma Aldrich) and phosphatase inhibitor cocktail (Sigma Aldrich) on ice until the cross-linked chromatin DNA was sheared to an average length of ∼500 bp. The samples were precleaned with beads and aliquoted for incubation with anti-ChREBP (Novus Biologicals, NB400-135), anti-FOXO1 (Abcam, Ab39670), or anti-MED1 (Bethyl Laboratories, A300-793A) at 4 °C for 16 h. The immunoprecipitated DNA complex was incubated with proteinase-K (Sigma Aldrich) mix at 65 °C for 12 h, extracted with phenol-chloroform, and precipitated with ethanol. Next, 1% of starting chromatin input values or cycle threshold (*C*_t_) values were used for normalization.

### 2.6 Histology, glycogen quantification, and G6PC activity assay

Liver tissues were fixed in formalin and subjected to standard H&E staining and PAS staining at the Albert Einstein College of Medicine Histology and Comparative Pathology Core. Glycogen levels in frozen liver tissues were measured by using a Glycogen assay kit II (Abcam, ab169558). For G6PC activity measurement, a G6PC activity assay kit (University of Buffalo BMR, E−120) was used.

### 2.7 RNA-seq

Total isolated RNA samples were sent to Novogene Corporation for genome-wide mRNA sequencing and followed the company's standard protocol in generating 150 bp cDNA fragments, paired and two-ended reads, and 30, 000, 000 fragments/library construction for an Illumina machine. The raw sequencing reads were trimmed using Trim Galore with the stringency 5 option. Differential expression was performed with DESeq2 [36] using transcript-level counts estimated by Kallisto [37]. A gene ontology analysis was performed using the DAVID functional annotation tool [38].

The RNA-seq data are publicly available at the GEO repository (accession number: GSE165033).

### 2.8 Luciferase reporter assay

Luciferase reporter assay was performed as previously described [39]. Briefly, isolated primary hepatocytes from 12-week-old male *Med13*^fl/fl^ or MED13-LKO mice were seeded and cultured at a density of 2 × 10^5^ per well in 12-well collagen-coated plates in DMEM with 10% FBS. The same day, the *Med13*^fl/fl^ cells were transduced with either adenovirus (Ad)-GFP or Ad-Cre. After an overnight culture, the primary hepatocytes were co-transfected with plasmids of pcDNA3 (vector), pcDNA-*Foxo1*, pGL4-*G6pc*-Luc, and Renilla-Luc (control) for FOXO1-induced *G6pc* promoter activity or pcDNA-*Chrebp-β* and pGL4-3xChoRE-Luc for ChREBP-β-induced ChoRE activity. The cells were then collected 24 h post-transfection for luciferase assays. The firefly luciferase activity of each sample was normalized by the renilla activity. The 3xChoRE sequence was 5′-AACATGCGCTGCAGGCATGTTCTTCCCATACCACGAGGTGGGAGGCTGTCCTAGACTAGTCACGTGTTCTACACGTGTT.

### 2.9 Statistical analysis

Data are presented as the mean ± SEM unless otherwise noted and compared between two groups using Student's *t*-test. A two-sided *p* < 0.05 was considered statistically significant. One-way ANOVA is also used as noted in the figure legends, followed by post hoc analysis for comparisons between individual groups. Identical letters in figures indicate datasets that are not statistically significant (*p* > 0.05).

## 3 Results

### 3.1 Liver-specific knockout of MED13 did not affect insulin sensitivity

**Figure 1:**
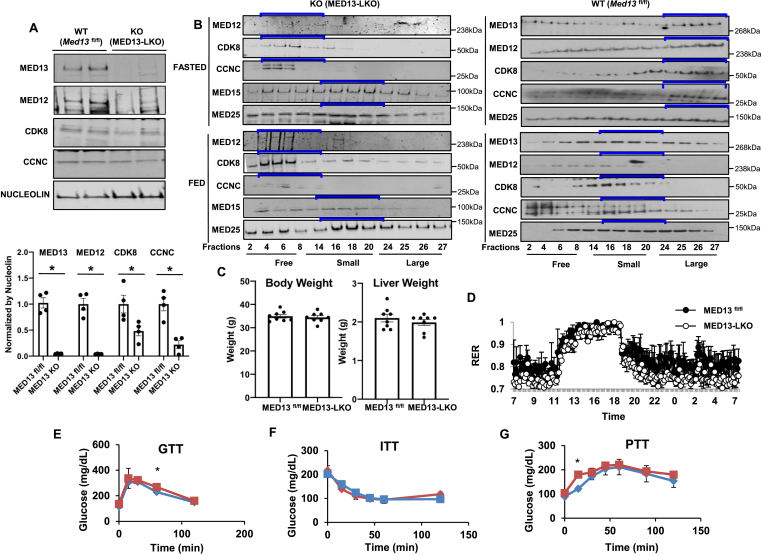
**Glucose metabolic phenotype of the MED13-LKO mice.** Twelve-week-old male *Med13*^*fl/*fl^ and MED13-LKO mice were fed normal chow (LabDiet #5053) ad libitum as described in Methods (n = 8 per group). **A)** Representative Western blotting images of liver nuclear proteins (5 separate experiments performed) and relative densitometric analysis of the mediator complex kinase module subunits. **B)** Glycerol gradient column separation and shift of kinase module subunits to the size that correlates with different complexes shown on gels. Fractions 2–8 represent free subunits, 14–20 represent small complexes, and 24–27 represent large complexes according to the size standards. Five livers of MED13-LKO (left panel) or *Med13*^*fl/*fl^ (right panel) mice that were fasted or fed with normal chow and 30% sucrose containing water were pooled and the same amount of liver protein lysate was loaded onto each column as described in Methods. **C)** Body weight and liver weight were measured in 12-week-old *Med13*^*fl/*fl^ and MED13-LKO mice after 4-h normal chow ad libitum feeding. **D)** Respiratory exchange ratio (RER) over a 24-h period. **E)** Glucose tolerance test, **F)** insulin tolerance test, and **G)** pyruvate tolerance test for *Med13*^*fl/*fl^ (red) and MED13-LKO (blue) mice were performed as described in Methods (n = 8 per group). *P* values were determined by Student's *t-*test (*p* < 0.05). Values are the mean ± SEM.

We recently reported that the kinase module subunits MED13, MED12, CDK8, and CCNC in the fed state dissociate from the large Mediator complex (∼1,200 kDa) to generate the small/core Mediator complex (∼600 kDa) with the subsequent degradation of the kinase module proteins [27]. Since MED13 directly links the kinase module to the core Mediator complex [40], loss of MED13 will essentially convert the large Mediator complex into the small Mediator complex. To understand the biological significance of fasting/feeding-regulated conversion of the Mediator complexes, we generated hepatocyte-selective MED13 knockout (MED13-LKO) mice by crossing *Med13*^*fl*/fl^ mice with *Alb-*Cre mice. As expected, compared with controls (*Med13*^fl/fl^), the MED13-LKO mice displayed a near complete loss of MED13 and MED12 with an approximately 40% reduction in CDK8 and CCNC proteins (Figure 1A).

To determine the assembly state of the Mediator complex, we utilized glycerol gradient fractionation to separate different complexes based on the molecular size differences, the small Mediator complex (∼600 kDa) from the large Mediator complex (∼1.2 MDa). As we previously reported [27], in the fasted state, Mediator subunits in nuclear extracts of livers from control, in this case *Med13*^*fl*/fl^, mice migrated on glycerol gradients as a high molecular weight structure in the ∼1 MDa range (Figure 1B, right panel). However, a significant fraction of liver Mediator subunits migrated to the ∼600 kDa range following 4 h of feeding due to the dissociation of the kinase module. As expected, liver nuclear extracts of fasted MED13-LKO had a very low level of MED12 but the migration of CDK8 and CCNC to low molecular weight proteins range was indicative of their dissociation from MED12 (Figure 1B, left panel) and this was confirmed by co-immunoprecipitation in our previous study [27]. Despite the dissociation and loss of the kinase module subunits, the small/core Mediator complex remained intact as observed by MED15 and MED25 enrichment in the ∼600 kDa range. Moreover, MED13 knockout did not cause additional changes in the Mediator subunit assembly state following 4 h of feeding.

To determine whether liver MED13 deficiency and loss of the Mediator kinase module affected whole body metabolic homeostasis, we compared the changes in body weight, liver weight, respiratory exchange ratio (RER), glucose tolerance (GTT), insulin tolerance (ITT), and pyruvate tolerance (PTT) between the control *Med13*^*fl*/fl^ and MED13-LKO mice (Figure 1C–G). In all of these metabolic assessments, there were no apparent differences between the *Med13*^*fl*/fl^ and MED13-LKO mice.

### 3.2 Liver-specific knockout of MED13 significantly reduced inducible gene expression

**Figure 2:**
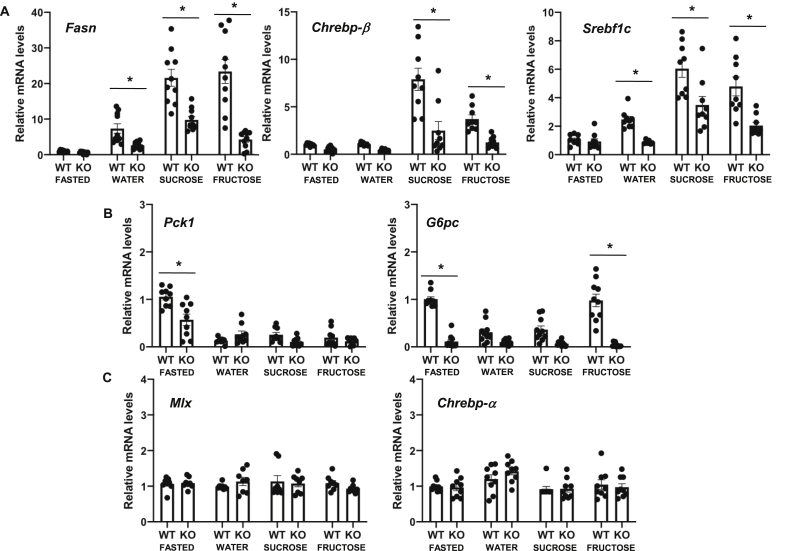
**Inducible gene expression was suppressed in MED13-LKO mice under different carbohydrate-containing feedings**. FASTED for 16 h fasted with water, WATER for normal chow supplemented with water, SUCROSE for normal chow supplemented with 30% sucrose containing water, and FRUCTOSE for normal chow supplemented with 30% fructose containing water. **A, B, and C)** Real-time quantitative RT-PCR (qRT-PCR) of lipogenic genes (*Fasn*, *Chrebp*-*β*, and *Srebf1c*), gluconeogenic genes (*G6pc* and *Pck1*), and constitutively active and unaffected by nutrient signal genes (*Mlx* and *Chrebp-α*) performed in livers of the *Med13*^*fl/*fl^ (WT) or MED13-LKO (KO) mice (n = 9 per group, 3 separate experiments were conducted). *P* values were determined by Student's *t-*test (*p* < 0.05). Values are the mean ± SEM.

To explore the kinase module's role in regulating metabolic gene expression, we assessed the feeding induction of lipogenic gene expression using qRT-PCR (Figure 2). *Fasn*, *Chrebp*-*β*, and *Srebf1c* mRNA were induced in the control *Med13*^fl/fl^ mice livers following 4 h of feeding with mouse chow only (Figure 2A). Supplementation of drinking water during the 4 h feeding period with sucrose or fructose further enhanced lipogenic gene expression. In contrast, the feeding induction of these genes with supplementation of sucrose and fructose was significantly suppressed in the MED13-LKO mice livers. In contrast to lipogenic genes that were induced in the fed state, the canonical fasting-induced gluconeogenic genes (*Pck1* and *G6pc*) were elevated in the fasted state and suppressed following feeding with chow and when supplemented with sucrose in the control *Med13*^*fl*/fl^ mice livers (Figure 2B). However, feeding with fructose suppressed *Pck1* mRNA but not *G6pc*, which remained elevated (Figure 2B). The MED13-LKO livers suppressed *G6pc* mRNA even in the presence of fructose.

To determine whether MED13 deficiency affected non-nutrient regulated genes in the liver, *Mlx* and *Chrebp-α* mRNA expression levels were measured in the *Med13*^fl/fl^ and MED13-LKO mice under these dietary conditions from the same mice (Figure 2C). There was no significant difference in the expression of these genes. Similarly, other constitutively expressed liver genes such as *Lxr-α* and *Usf1* were unaffected by MED13 deficiency (Fig. S1). In addition, genes that regulate glycogen synthesis or breakdown, *Gbe*, *Phkb*, and *Gs*, showed no significant changes with changes in nutritional states or MED13 deficiency (Fig. S1). The relative effect of MED13 deficiency on nutrient-regulated liver genes but not constitutive gene expression was confirmed by genome-wide RNA-seq analyses for the sucrose- and fructose-fed mice (Fig. S2). Based on these data, we conclude that the kinase module primarily regulates inducible gene expression but is not essential for basal gene expression in the liver. Moreover, MED13 deficiency suppressed *G6pc* gene expression even in the fasting and fructose-fed conditions where G6pc expression levels should be highly induced.

### 3.3 Fructose suppressed liver glycogen accumulation in the fed state

**Figure 3:**
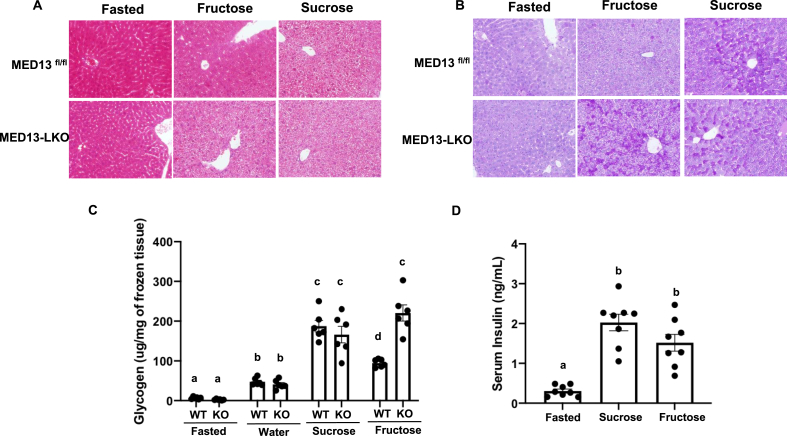
**Glycogen accumulation was observed in MED13-LKO liver under fructose feeding**. **A)** Representative H&E stained liver sections of *Med13*^*fl/*fl^ and MED13-LKO after overnight fasting followed by chow feeding supplemented with 30% fructose containing water for 4 h. **B)** Representative periodic acid Schiff (PAS)-stained liver sections of the *Med3*^*fl/*fl^ and MED13-LKO mice. **C)** Hepatic glycogen measurement from the *Med13*^*fl/*fl^ and MED13-LKO mice under fasted or ad libitum fed supplemented with either water or water with 30% fructose or 30% sucrose conditions. **D)** Serum insulin levels of WT mice after feeding (n = 8 per group). Statistical significance was determined by one-way ANOVA followed by the Turkey-Kramer multiple comparisons test. Bars with the same letter are not significantly different.

The hepatic production and release of glucose is a complex process that includes *de novo* formation of glucose by gluconeogenesis and breakdown of glycogen through glycogenolysis [41,42]. Previous studies also indicated that chronic fructose feeding results in elevated gluconeogenesis due to increased expression of *G6pc*, which encodes the catalytic subunit of G6PC (or G6Pase) [43,44]. Because G6Pase converts glucose 6-phosphate to glucose in the last step for both gluconeogenesis and glycogenolysis, we assessed the effect of acute fructose and MED13 deficiency on glycogen repletion. As expected, in the fasted state in the control *Med13*^fl/fl^ and MED13-LKO mice, the glycogen levels were relatively low as assessed by H&E staining, periodic acid Schiff (PAS) labeling, and quantification of liver glycogen (Figure 3A–C). When the mice were fed with sucrose supplementation, there was a marked increase in liver glycogen content that was not significantly different between the control *Med13*^fl/fl^ and MED13-LKO mice. However, supplementation with fructose in the drinking water resulted in a reduction in glycogen accumulation compared to sucrose supplementation in the control *Med13*^fl/fl^ mice but that was not observed in the MED13-LKO mice livers. The reduction in the glycogen levels with fructose supplementation in the control *Med13*^fl/fl^ mice was not a result of altered insulin secretion as there was no statistically significant difference in insulin levels (Figure 3D), although there was a trend of lower insulin levels in the fructose-supplemented mice during early periods of the post-prandial time course (Fig. S3A). The post-prandial rise in glucose also trended to be lower in the fructose-supplemented mice but this was not statistically significant (Fig. S3B).

### 3.4 Overexpression of G6PC restored fructose-caused reduction of glycogen accumulation in MED13-LKO mice

**Figure 4:**
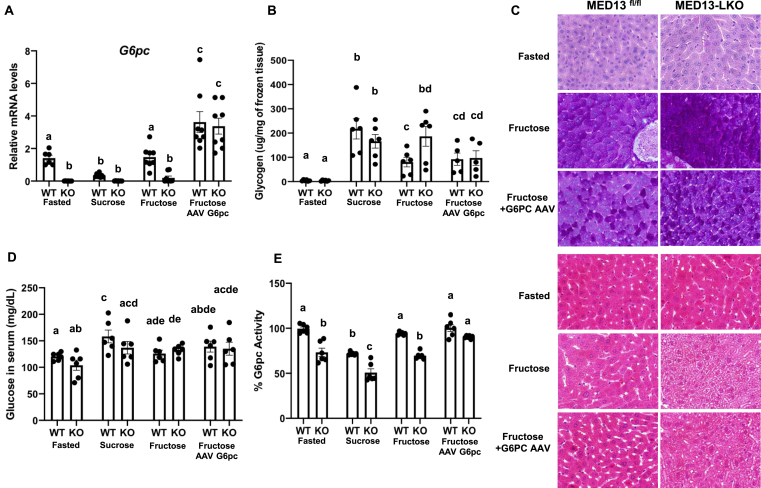
**Overexpression of *G6pc* decreased glycogen accumulation in MED13-LKO. A)***G6pc* gene expression in both *Med13*^fl/fl^ and MED13-LKO mice liver was measured by qRT-PCR 10 days after AAV-*Tbg-GFP* or AAV-*Tbg-G6pc* injections. **B)** Hepatic glycogen measurement, **C)** H & E staining and PAS staining in liver sections and hepatic glycogen levels, **D)** changes in serum glucose levels, and **E)** % changes in G6pc activity (n = 6) were measured as described in Methods (n = 8 per group). Identical letters indicate values that are not statistically different from each other (*p* > 0.05). One-way ANOVA followed by the Turkey-Kramer multiple comparisons test.

If the inability of fructose to suppress *G6pc* gene expression is the causative pathway preventing glycogen accumulation in the fed state, then constitutive *G6pc* expression should reverse the ability of the MED13-LKO to restore glycogen. To this end, we infected the control *Med13*^*fl*/fl^ and MED13-LKO mice with adeno-associated viruses (AAV) (AAV*-Tbg-GFP* or AAV*-Tbg-G6pc*) to overexpress G6Pase specifically in hepatocytes under the control of the *Tbg* promoter, which is not regulated by nutrients. As shown in Figure 2, the *G6pc* mRNA level was elevated in the fasted state and suppressed when the control *Med13*^*fl*/fl^ mice were fed with sucrose supplementation (Figure 4A). However, *G6pc* mRNA failed to decrease in the fructose-fed control mice whereas *G6pc* was suppressed in both the fasted and fructose-fed states in the MED13-LKO mice livers. In contrast, *G6pc* mRNA remained elevated approximately 2-fold over the fasted state in both the control *Med13*^fl/fl^ and MED13-LKO mice following *G6pc* overexpression with fructose supplementation.

Glycogen content increased in the sucrose-supplemented control *Med13*^*fl*/fl^ mice and was suppressed in the fructose-supplemented mice whereas the MED13-LKO mice were able to accumulate glycogen following fructose supplementation (Figure 4B and C). In contrast, the fructose suppression of liver glycogen accumulation was not significantly different between the control *Med13*^*fl*/fl^ and MED13-LKO mice when *G6pc* was overexpressed. As a control, serum glucose levels were measured that increased in the post-prandial states but were not significantly different in the *Med13*^*fl*/fl^ and MED13-LKO mice whether fed with sucrose or fructose supplementation or by overexpression of *G6pc* (Figure 4D). G6Pase enzymatic activities were measured and the levels were in agreement with the *G6pc* mRNA levels (Figure 4E). These data demonstrate that constitutive overexpression of G6PC is sufficient to prevent liver glycogen restoration in the fed state.

### 3.5 MED13 deficiency did not inhibit proximal insulin signaling

**Figure 5:**
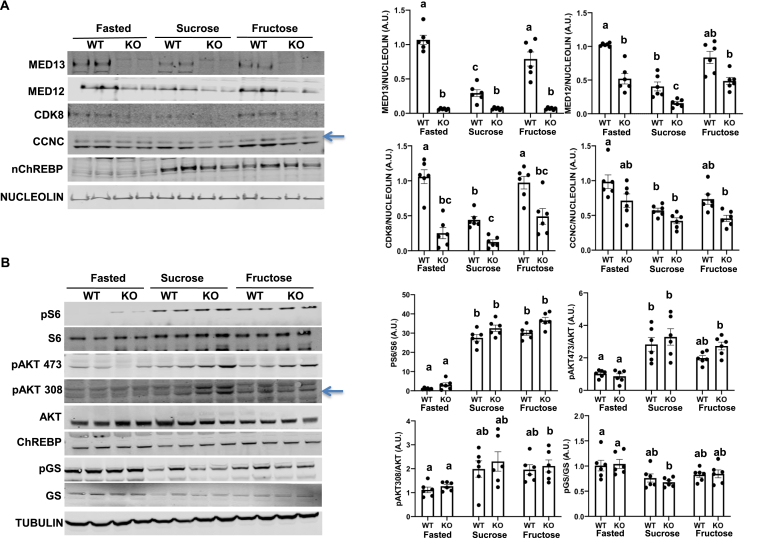
**Fructose supplementation reduced insulin signaling and suppressed the downregulation of the Mediator kinase module. A)** Western blotting and relative densitometric analysis of hepatic nuclear MED13, MED12, CDK8, CCNC, nuclear ChREBP, and nucleolin loading control. **B)** Western blotting and relative densitometric of total liver including nuclear and cytosolic fractions (n = 6 per group, representative duplicate from 3 separate experiments shown on gels). *P* values were determined by Student's *t-*test (*p* < 0.05). Values are the mean ± SEM. Identical letters indicate values that are not statistically different from each other (*p* > 0.05). Multiple sample comparisons were determined by one-way ANOVA followed by the Turkey-Kramer multiple comparisons test.

The Mediator complex kinase module subunits (MED13, MED12, CDK8, and CCNC) undergo degradation in the fed state [27,33]. Consistent with these findings, Western blotting analyses of the kinase module subunits displayed a reduction in these proteins in the control *Med13*^fl/fl^ mice following feeding with chow plus sucrose compared to fasted mice livers (Figure 5A). In contrast, when the mice were fed with fructose-supplemented water, there was no significant decrease in the kinase module subunit proteins. The transcription factor ChREBP is known to undergo nuclear translocation from the cytoplasm in the fed state [19], and we observed a similar extent of ChREBP nuclear translocation in both the sucrose and fructose-supplemented fed mice. Moreover, MED13 deficiency had no significant effect on ChREBP nuclear translocation (Figure 5A), with no apparent change in the total levels of ChREBP protein (Figure 5B). Our previous studies suggested that kinase module protein reduction depends on activation of the mTORC1 pathway [27,33]. There was no significant difference in S6, AKT, or glycogen synthase (GS) phosphorylation states in the control *Med13*^fl/fl^ mice induced by either sucrose or fructose-supplemented feeding (Figure 5B). Consistent with previous reports [63,65], feeding resulted in a transient increase in FOXO1 phosphorylation but with a slower and more sustained decrease in GS phosphorylation (Figs. S5A–C). However, there was no significant difference between the effect of sucrose or fructose supplementation, consistent with the similar levels of insulin secretion (Figure 3 and Fig. S3).

### *3.6* MED13 deficiency altered ChREBP binding to the *G6pc* promoter

**Figure 6:**
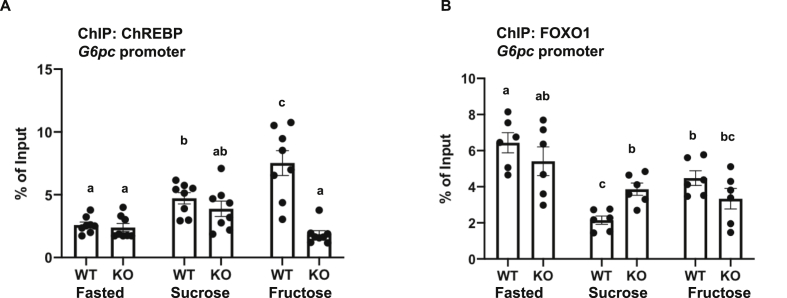
**Binding of ChREBP and FOXO1 transfactors on *G6pc* promoter in MED13-LKO mice.** ChIP-qPCR was performed to analyze the enrichment for transfactors in the *G6pc* promoter region. **A)** ChREBP-specific antibody was used to pull down sonicated liver chromatin of the *Med13*^fl/fl^ and *MED13-LKO* mice (n = 6). Precipitated DNA enrichment is shown as a percentage of input DNA, with average Ct values of 31.2 (Fasted WT), 29.65 (Fasted KO), 30.38 (Sucrose WT), 29.3 (Sucrose KO), and 30.0 (Fructose WT). **B)** FOXO1-specific antibody was used to pull down sonicated liver chromatin of the *Med13*^fl/fl^ and *MED13-LKO* mice (n = 6). Precipitated DNA enrichment is shown as a percentage of input DNA, with average Ct values of 32.4 (Fasted WT), 31.5 (Fasted KO), 33.8 (Sucrose WT), 31.9 (Sucrose KO), 34.1 (Fructose WT), and 31.8 (Fructose KO). Non-specific IgG was used as a control, which displayed Ct values > 38 or were undetectable. A promoter set of the non-specific upstream region was also used as a control, which showed Ct value > 38 or was undetectable. *P* values were determined by one-way ANOVA followed by the Turkey-Kramer multiple comparisons test. Error bars indicate the mean ± SEM. Bars with different letters are significantly different from each other (*p* < 0.05).

In the fasted state, *G6pc* expression is transactivated by FOXO1, and in the fed state, FOXO1 is inactivated by acetylation and nuclear-exported due to AKT-dependent phosphorylation [45]. The *G6pc* gene also contains a ChREBP-responsive cis-DNA element and fructose is a potent activator of ChREBP, which has been reported to be the basis for the elevated activation of *G6pc* expression by fructose [7]. To examine the basis for MED13 regulation of *G6pc*, we first performed chromatin immuno-precipitation coupled with quantitative PCR (ChIP-qPCR) to determine FOXO1 and ChREBP binding on the *G6pc* gene promoter (Figure 6A and B). In the fasted state, there was a relatively low level of ChREBP binding to *G6pc*, which was not different between the control *Med13*^fl/fl^ and MED13-LKO mice livers (Figure 6A). Surprisingly, in the control livers, ChREBP binding increased following feeding with sucrose supplementation, although *G6pc* gene expression was substantially suppressed. Similarly, the control mice fed with fructose supplementation also displayed increased ChREBP binding, but more importantly, fructose supplementation did not induce ChREBP binding to the *G6pc* promoter in the MED13-LKO mice, suggesting that the interaction of ChREBP with the *G6pc* gene promoter is MED13-dependent. In contrast, the binding of FOXO1 to the *G6pc* gene in the fasted state was not disrupted in the MED13-LKO mice (Figure 6B). Moreover, under sucrose supplementation, FOXO1 binding to the *G6pc* gene promoter was downregulated in both the *Med13*^fl/fl^ and MED13-LKO mice.

To confirm that the Mediator complex remains able to bind to the *G6pc* promoter in either the presence or absence of MED13, we also performed ChIP-qPCR using a well-tested antibody against the core Mediator subunit MED1 in the control and MED13-LKO mice. As shown in Fig. S4A, MED13 deficiency had no effect on MED1 occupancy on the *G6pc* promoter. MED1 occupancy was somewhat reduced in the fed state with sucrose but not in the fed state with fructose, indicating that Mediator binding was not the primary reason for the nutrient regulation of *G6pc* gene expression. As an additional control, we also assessed ChREBP binding to the *Fasn* promoter (Fig. S4B). As expected, ChREBP occupancy increased in the sucrose- and fructose-fed state in the control mice that was reduced in the MED13-LKO mice livers.

**Figure 7:**
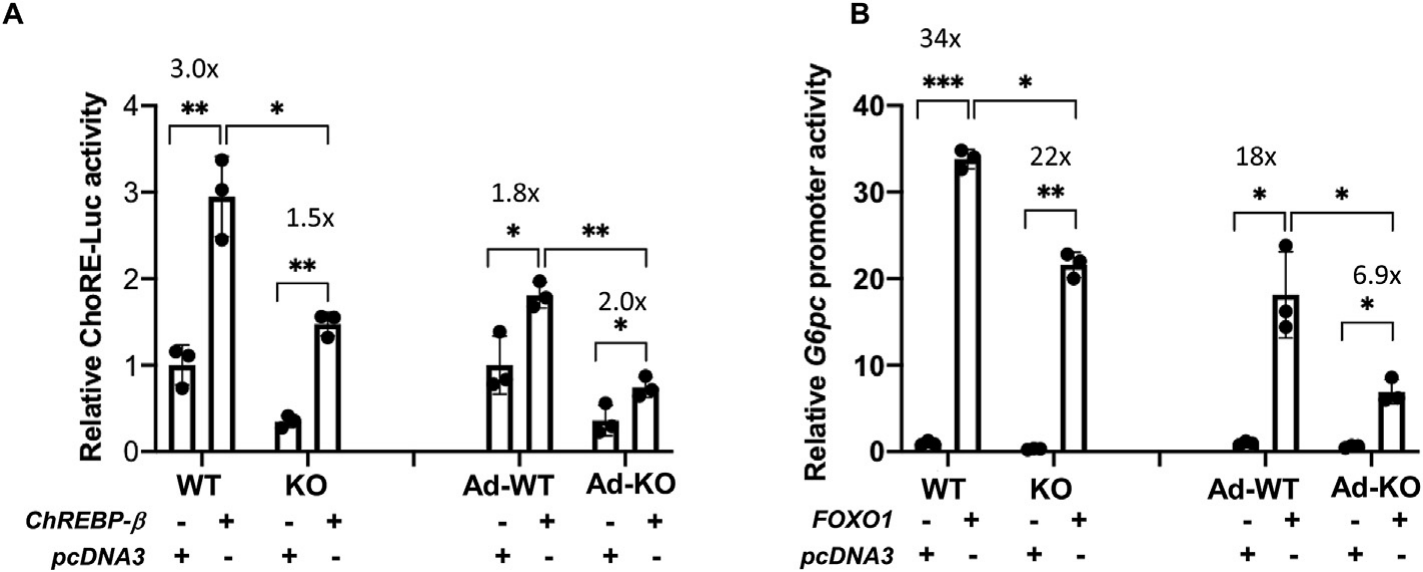
**Luciferase reporter assay of ChREBP and FOXO1 transfactors in MED13-LKO mice.** Isolated primary hepatocytes from adenovirus (Ad)-GFP (Ad-WT) or Ad-Cre (Ad-KO) transduced *Med13*^fl/f^^l^ (WT) or *Med13*^fl/fl^*Alb*-Cre (KO) mice were used. **A)** Luciferase reporter assays for the effects of ChREBP-β on ChoRE activity in either *Med13*^fl/fl^ (WT and Ad-WT) or *Med13-LKO* (KO and Ad-KO) as indicated. **B)** Luciferase reporter assays for the effects of FOXO1 on *G6pc* promoter activity in indicated cells, either from *Med13*^fl/fl^ (WT and Ad-WT) or *Med13-LKO* (KO and Ad-KO). *P* values were determined by Student's *t-*test (*p* < 0.05). ∗*p* < 0.05, ∗∗*p* < 0.01, ∗∗∗*p* < 0.001, and n = 3. The values above each of the error bars indicate the fold stimulation.

Although DNA binding of regulatory factors is an important component of gene expression, this does not directly address functional transcriptional activity. Therefore, we next isolated primary hepatocytes from the control and MED13-LKO mice and co-transfected them with expression plasmids for ChREBP-β and a synthetic ChoRE-luciferase reporter, which only contains three copies of ChoRE (Figure 7A). As shown in the left bars, both the basal and ChREBP-β-induced ChoRE promoter activity of the ChREBP reporter was significantly suppressed by MED13 deficiency. To eliminate any potential development effects in the MED13-LKO hepatocytes, we also isolated primary hepatocytes from the *Med13*^*fl*/fl^ mice, which were then infected with adenovirus expressing GFP (Ad-GFP) or Cre (Ad-Cre) with the expression plasmids for ChREBP-β and the ChoRE-luciferase reporter (Figure 7A, right bars). We again observed a significant suppression of basal and ChREBP-β-stimulated transcriptional activity in the acute MED13 knockout hepatocytes. Similarly, examination of the FOXO1 transcriptional activity using the proximal *G6pc* promoter also demonstrated a reduced ability of FOXO1 to transactivate *G6pc* reporter in the MED13 knockout hepatocytes (Figure 7B). In this case, there was no significant change in basal reporter activity only on the FOXO1-induced transcriptional activation.

To further examine whether the fructose regulation of *G6pc* expression was cell autonomous and independent of systemic metabolic signaling, we isolated primary hepatocytes from the control and MED13-LKO mice (Fig. S6A). Fructose treatment alone increased the expression of *G6pc*, *Fasn*, and *Chrebp-β* mRNA in the control cells compared to primary hepatocytes maintained under regular tissue culture conditions (Figs. S6B–D). Importantly and consistent with the effect of fructose supplementation *in* *vivo* (Figure 2), in the MED13-deficient hepatocytes, both the basal and fructose-stimulated increase in *G6pc*, *Fasn* and, *Chrebp-β* mRNA was markedly suppressed. In contrast, constitutively expressed genes *Mlx*, *Foxo1*, and *Usf1* that are not nutrient-regulated *invivo* were neither affected by fructose treatment nor suppressed in the MED13 knockout hepatocytes (Figs. S6E–G). Together, these data support the hypothesis that the kinase module of the Mediator complex plays an important regulator role in controlling transcription factor DNA occupancy and transcriptional activity in liver hepatocytes that are i) transfactor-specific, ii) nutrient-specific, and iii) target gene-specific.

## 4 Discussion

The integration of gene transcription is coordinated by multiple regulator factors of which the Mediator plays an essential role [46,47]. In particular, elements of the Mediator kinase module have been observed to play various roles in a tissue/cell-type context-dependent manner. For example, CDK8 was found to function both as a proto-oncogene and tumor suppressor [[48], [49], [50]]. In *Drosophila* and mice, CDK8 was also observed to regulate nuclear SREBP-1c degradation and independently CCNC was reported to regulate mitochondrial fission [51,52]. Deletion of MED13 in mouse cardiomyocytes enhanced obesity in response to a high-fat diet and exacerbated metabolic syndrome due to the inhibition of a cardiomyocyte secretory factor [53]. In contrast, MED13 deficiency in skeletal muscle increased insulin sensitivity and protected mice against high-fat diet-induced hepatic steatosis [54].

In the liver we recently observed that the large Mediator complex predominantly exists in the fasted state but that a substantial proportion converts to the small/core Mediator complex in the fed state due to both dissociation and subsequent degradation of the kinase module subunits, MED13, MED12, CDK8, and CCNC [27,33]. Of the approximately 12,000 protein-encoding genes expressed in the liver, approximately 3000 undergo regulated changes between the fasted and fed states [55]. In particular, among the most dynamically modulated genes during the fasted/fed transitions are those regulating the gluconeogenic and lipogenic pathways. In this regard, several studies have demonstrated that dietary fructose has significant patho-metabolic properties and may be an important contributor to the development of fasting hyperglycemia, insulin resistance, obesity, and non-alcoholic fatty liver disease [56]. However, the mechanisms of fructose action and downstream physiologic responses are highly complex and poorly understood. For example, fructose has been reported to induce [57] and inhibit beta cell insulin secretion [[58], [59], [60]]. In this regard, we have observed a trend for fructose to reduce insulin secretion compared to sucrose but this was not statistically significant and in any case, insulin levels were within the post-prandial physiologic range with no deleterious effect on proximal insulin-signaling events.

As shown in Fig. S4, we observed no significant difference in the upregulation of blood glucose levels and serum insulin levels in the mice that were under the sucrose water or fructose water supplementation conditions in all of the post-prandial time points measured. Consistent with several recent studies [6,7,61], fructose feeding also prevented the post-prandial decline in *G6pc* expression. Under the combination of a high-fat and high-fructose diet, chronic feeding elevated *G6pc* levels through transactivation by ChREBP-β [6]. CHREBP-α is activated in the fed state, which in turn transactivates ChREBP-β that is an important transfactor regulating lipogenic gene expression [62]. Although the *G6pc* gene is typically suppressed in the fed state, its distal promoter contains a ChREBP-binding element and can be transactivated by ChREBP transcription factors [7]. Our data indicate that the acute effect of fructose to prevent the suppression of *G6pc* is responsible for the inhibition of glycogen accumulation during the fasted to fed transition.

It should be noted that two previous studies also examined the effect of post-prandial fructose on liver glycogen repletion. Conlee et al. [64] provided gastric-infused glucose and fructose to rats following fasting but only observed a small increase of glycogen repletion to approximately 20% of normal liver glycogen levels in both cases. Similarly, Sato et al. [63] also used intragastric-delivered fructose in fasted mice and observed glycogen restoration to relatively normal fed liver glycogen levels. However, in this study, fructose prevented the decline in *G6pc* mRNA that occurs following feeding but with a reduction in G6PC activity. Other than the different methodologies used in these previous studies, the basis for the differences with our findings remains to be determined.

Nevertheless, at the molecular level, our data indicate that ChREBP occupies the *G6pc* promoter in the fed state with both sucrose and fructose supplementation. However, *G6pc* gene expression remains elevated only when fructose is present in the diet. Previously using FOXO1 knockout mice, fructose feeding was reported to induce *G6pc* gene expression independent of FOXO1 [7]. Consistent with these data, in both sucrose- and fructose-supplemented feeding, FOXO1 binding to the *G6pc* promoter was similarly reduced. An important difference between sucrose- and fructose-supplemented feeding is the molecular state of the Mediator complex. Feeding with sucrose shifts the large Mediator complex present in the fasted state to that of the small/core Mediator complex. However, in the presence of fructose, the Mediator complex primarily remains as the large Mediator complex. Assuming that the large Mediator complex is the major driver for the induction of gluconeogenic gene expression whereas the small/core Mediator is the major driver for lipogenic gene expression, this would account for ChREBP's ability to drive *G6pc* gene expression in the presence of fructose. Moreover, this would also account for the effect of MED13 deficiency to suppress *G6pc* gene expression in the fasted, sucrose-, and fructose-fed states. As the Mediator continuously undergoes dynamic oligomeric state reorganization, it is likely that this reflects many of the gene and cell context effects of Mediator complex-dependent gene expression. Future studies are needed to identify the specific mechanisms responsible for this unusual form of transcription specificity in the liver.

## Significance statement

The Mediator is a multisubunit protein complex that links transcription factors to RNA polymerase II and undergoes dynamic state reorganization between the small or core Mediator complex (26 subunits) and the large Mediator complex that also contains the kinase module subunits (MED13, MED12, CDK8, and CCNC). In the liver, we found that deletion of the MED13 subunit resulted in the constitutive formation of the small Mediator and the downregulation of nutrient-inducible gene expression. Fructose suppression of glycogen synthesis occurred due to increased *G6pc* expression and MED13 deficiency reversed the detrimental effect of fructose by inhibiting *G6pc*. These data provide direct evidence for the kinase module as a regulator of inducible gene expression and preventing fructose-mediated metabolic dysregulation in the liver.

## Figures and Tables

**Fig. s1:**
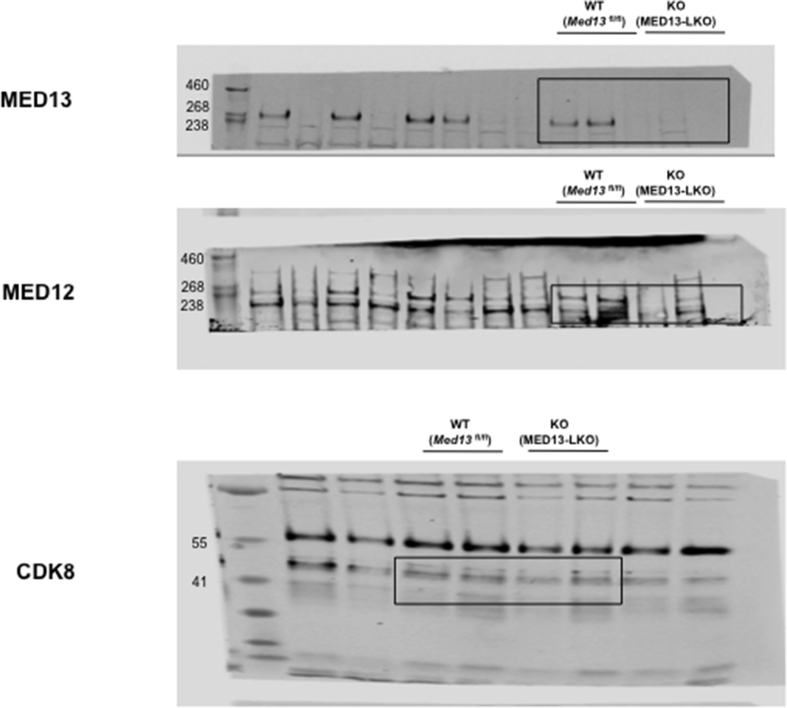
**Gene expression of nutrient-unresponsive genes.** The *Med13*^fl/fl^ control mice (WT) and MED13-LKO (KO) mice were fasted for 16 h overnight and sacrificed (fasted) or fed with water or fed with normal chow with supplementation of 30% sucrose or fructose water (sucrose, fructose). Real-time quantitative RT-PCR (qRT-PCR) analysis of glycogen synthesis or breakdown pathway-regulating genes (*Gbe*, *Phkb*, and *Gs*) and nutrient-unresponsive genes (*Lxr-α* and *Usf1*) was performed in livers of the *Med13*^fl/fl^ or MED13-LKO mice (n = 4 per group for glycogen-related genes and n = 3 for nutrient-unresponsive genes). No statistically significant differences between samples were found.

**Fig. s2:**
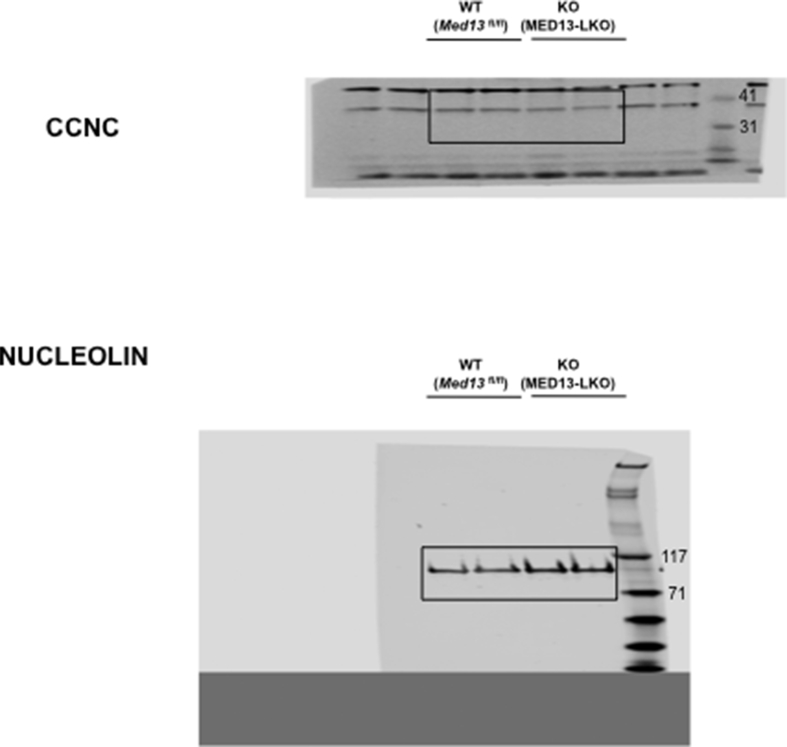
**Heatmap of RNA-seq reporting changes in hepatic genes in the *Med13***^***fl*/fl**^**and MED13-LKO mice under fed states. A)** Absolute of log2foldchange in gene expression for the *Med13*^*fl*/fl^ and MED13-LKO mice under feeding with 30% sucrose or fructose water supplementation compared with fasted liver samples. Genes with |log2foldchange| > 1 and padj (adjusted p value) < 0.05 in the MED13^fl/fl^ mice were selected as rows of the heatmap, and the intensity of the red color represents the magnitude of fold changes in either the up or down direction. **B)** Log2foldchange in gene expression for the *Med13*^*fl*/fl^ and MED13-LKO mice under feeding with 30% sucrose or fructose water supplementation compared with fasted liver samples. Genes with |log2foldchange| > 1 and padj (adjusted *p* value) < 0.05 in the *Med13*^*fl*/fl^ mice were selected as rows of the heatmap, and the intensity of the red color represents the magnitude of fold changes in either the up or down direction. **C)** Feeding induced and reduced gene populations in the *Med13*^fl/fl^ (blue) and MED13-LKO (orange) mice with sucrose or fructose water supplementation. Genes with padj <0.05 were counted. **D)** Gene ontology analysis of both upregulated and downregulated genes in the MED13-LKO mice compared to the *Med13*^fl/fl^ mice under fructose water supplementation. Genes with |log2foldchange| > 1 and padj (adjusted *p* value) < 0.05 were used as an input list to the DAVID functional annotation tool, and all of the genes detected in the experiment were used as background. Bar graph shows log10(padj) and the numbers beside the bars show the number of input genes in the GO term.

**Fig. s3:**
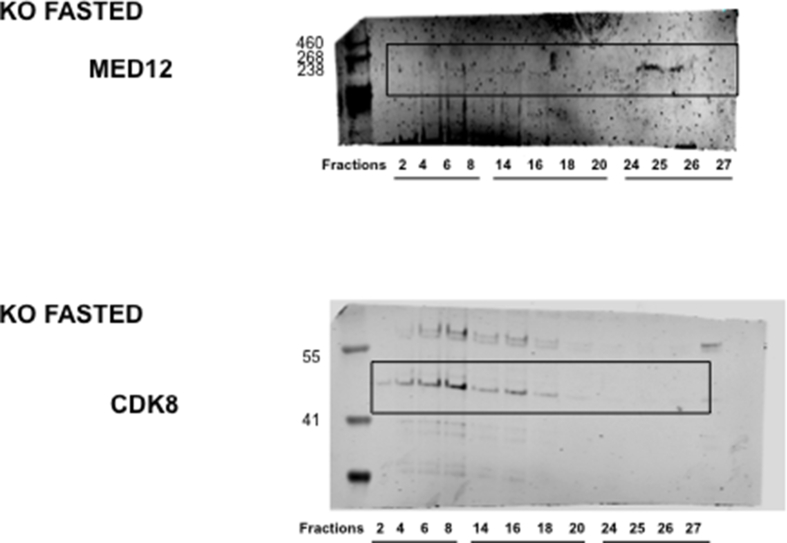
**Circulating glucose and insulin levels under different carbohydrates containing water supplementations and at different post-prandial time points.** Twelve-week-old C57BL/6J mice were fasted overnight and fed with normal chow supplemented with 30% sucrose or fructose in their drinking water. **A)** Blood glucose was measured at each post-prandial time point as indicated. **B)** Serum insulin was measured at each post-prandial time point as indicated. *P* values were determined by Student's *t-*test (*p* < 0.05). Values are the mean ± SEM.

**Fig. s4:**
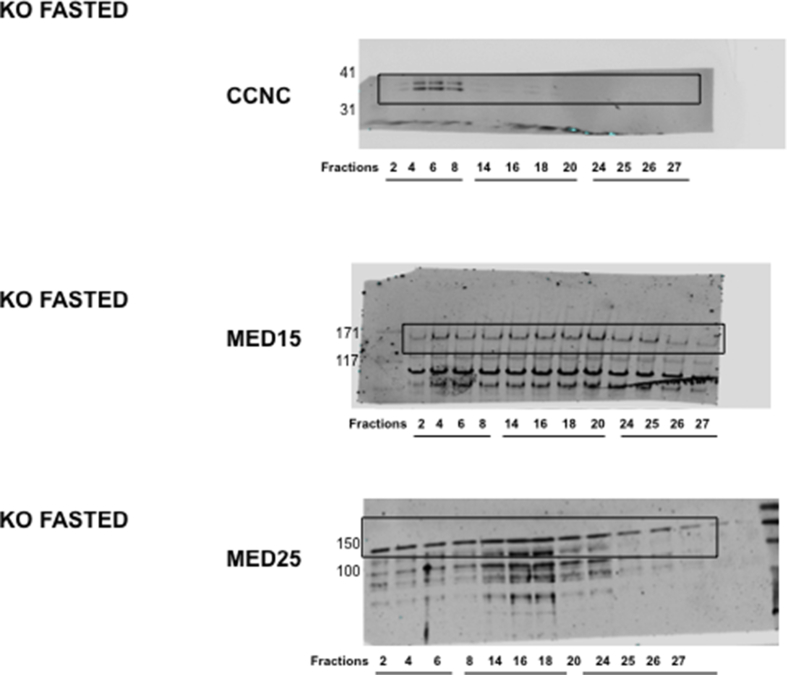
**Binding of MED1 on *G6pc* promoter and ChREBP transfactors on *Fasn* promoter in the MED13**^**fl/fl**^**(WT) and MED13-LKO (KO) mice.** ChIP-qPCR was performed to analyze the enrichment of the core mediator subunit MED1 on the *G6pc* promoter region and ChREBP transfactors on the *Fasn* promoter region. **A)** Anti-MED1 and **B)** anti-ChREBP were used to pull down sonicated liver chromatin of the *Med13*^*fl*/fl^ and MED13-LKO mice (n = 4). Precipitated DNA enrichment is shown as a percentage of input DNA. *P* values were determined by one-way ANOVA followed by the Turkey-Kramer multiple comparisons test. Error bars indicate the mean ± SEM. Bars with different letters are significantly different from each other (*p* < 0.05).

**Fig. s5:**
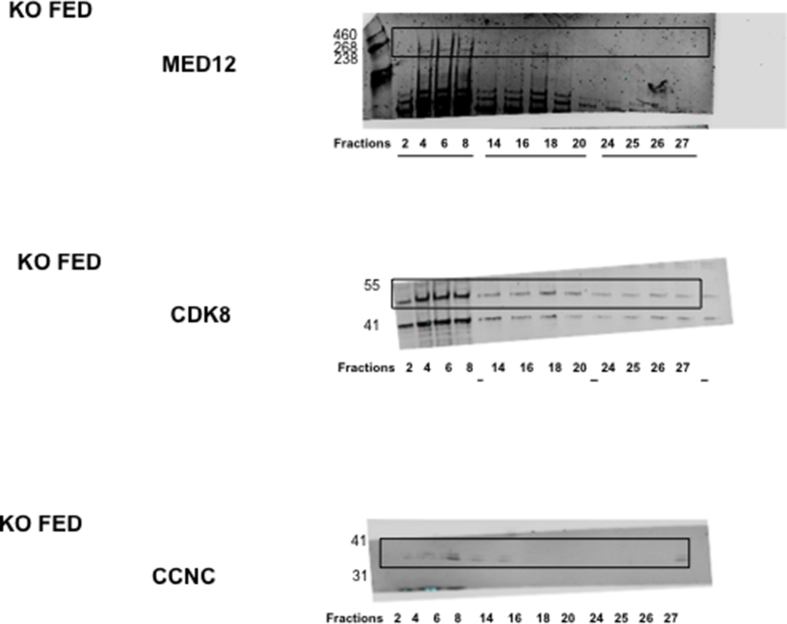
**FOXO1 and GS phosphorylation levels under different carbohydrates containing water at different feeding times.** Twelve-week-old C57BL/6J mice were fasted overnight and fed with normal chow supplemented with 30% sucrose or fructose in their drinking water. **A)** Representative Western blotting images of phosphorylated FOXO1 (S256), total FOXO1, phosphorylated GS (S641), and tubulin from two independent mouse livers. **B)** Relative densitometric analysis of pFOXO1/tubulin and **C)** pGS/tubulin is presented. *P* values were determined by one-way ANOVA followed by the Turkey-Kramer multiple comparisons test. Error bars indicate the mean ± SD. Bars with different letters are significantly different from each other (*p* < 0.05).

**Fig. s6:**
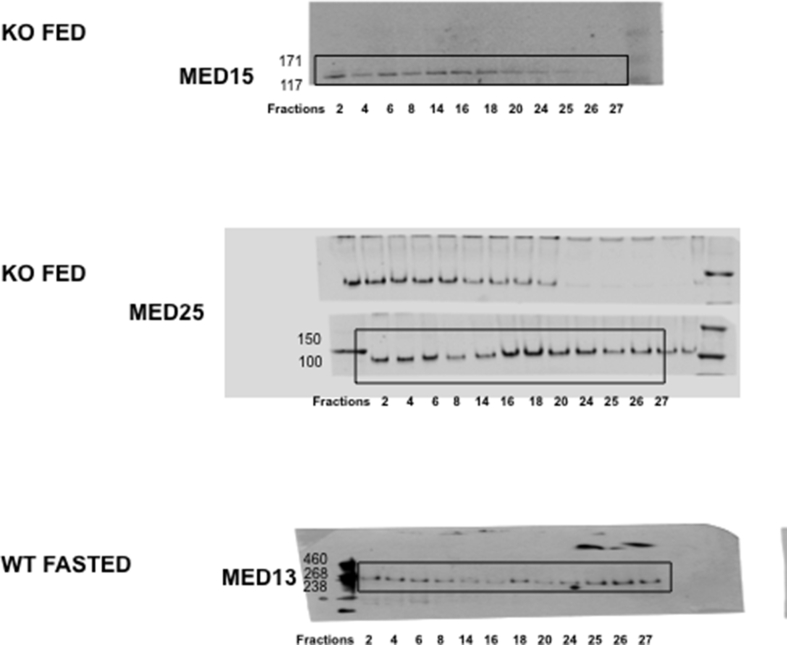
**Fructose induction of endogenous gene expression in primary hepatocytes isolated from the control and MED13-LKO mice.** Primary mouse hepatocytes were isolated from the WT (*Med13*^fl/fl^) or KO (*Med13*-LKO) mice. The cells were cultured on collagen-coated plates in DMEM without glucose (−) or with 45 mM of fructose (+) for 5 h. Cellular RNA was isolated and subjected to qRT-PCR for **A)***Med13*, **B)***G6pc*, **C)***Fasn*, **D)***Chrebp-β*, **E)***Mlx*, **F)***Foxo1*, and **G)***Usf1* mRNA. *P* values were determined by Student's *t-*test (p < 0.05). Values are the mean ± SD.

**Fig. s7:**
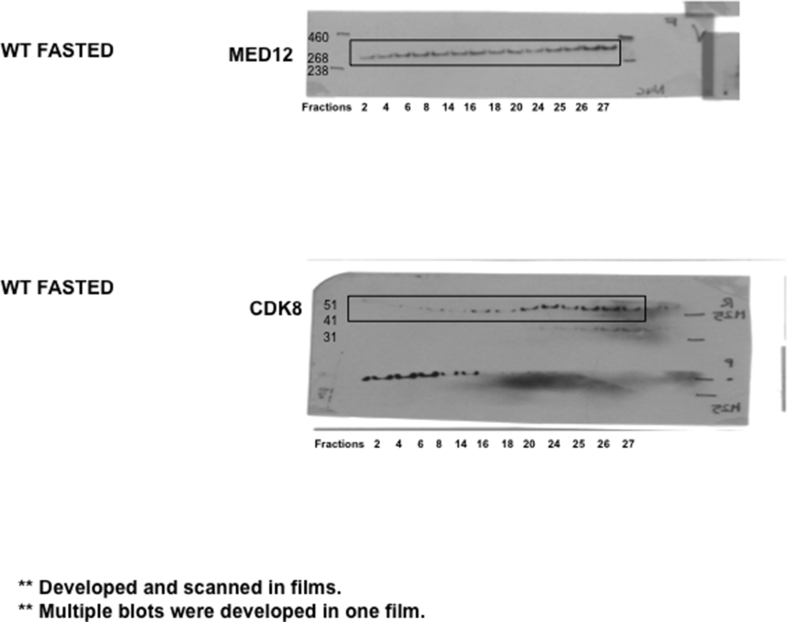


**Fig. s8:**
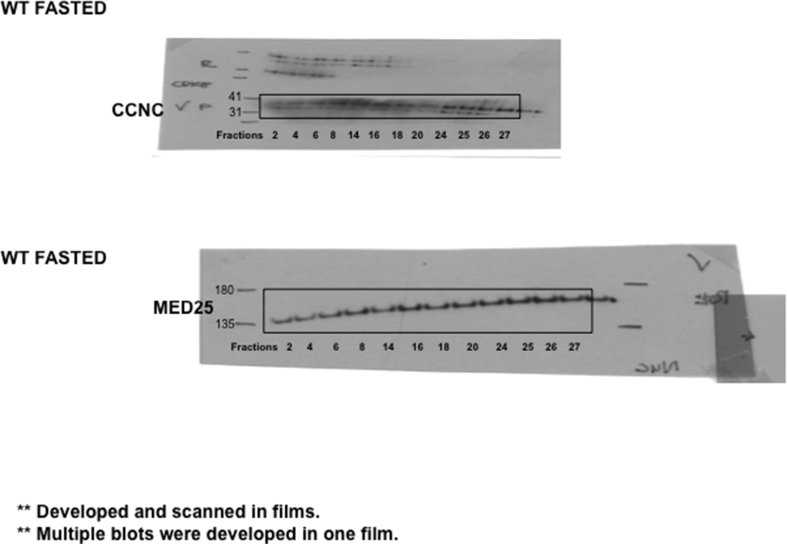


**Fig.s9:**
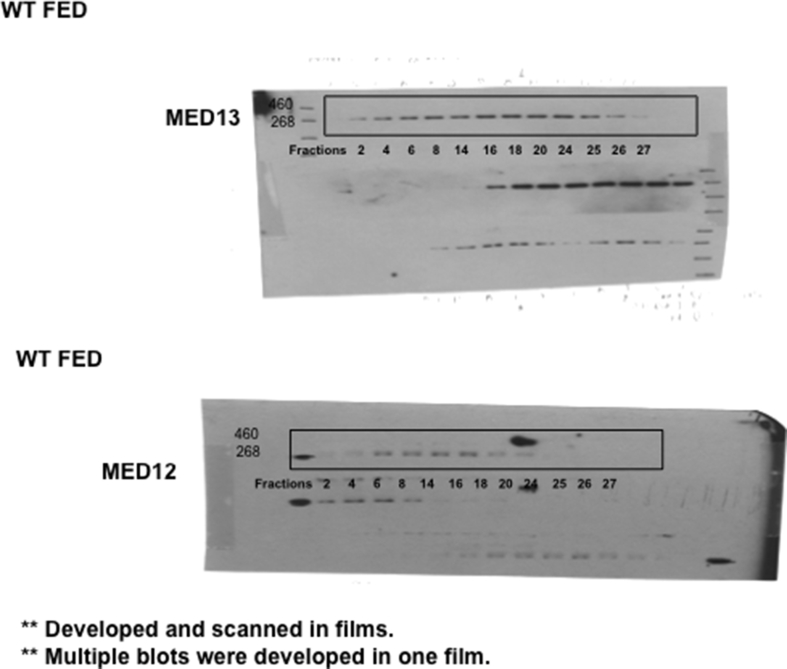


**Fig. s10:**
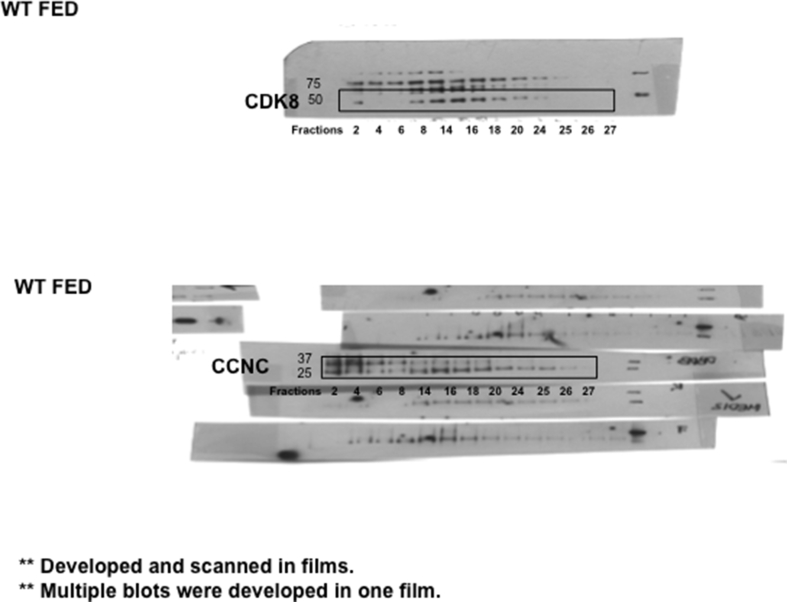


**Fig. s11:**
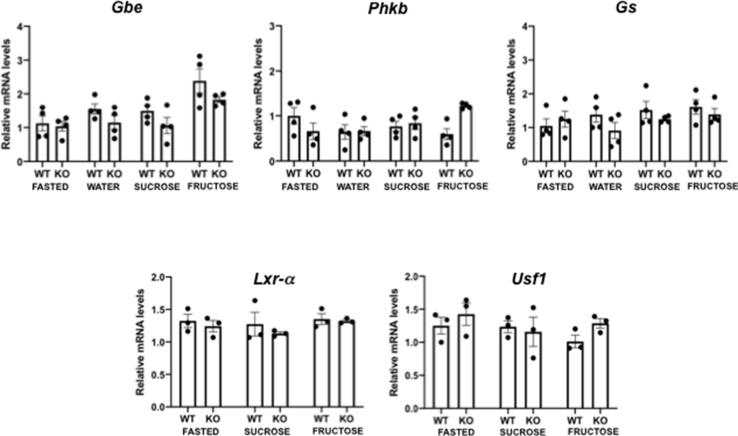


**Fig. s12:**
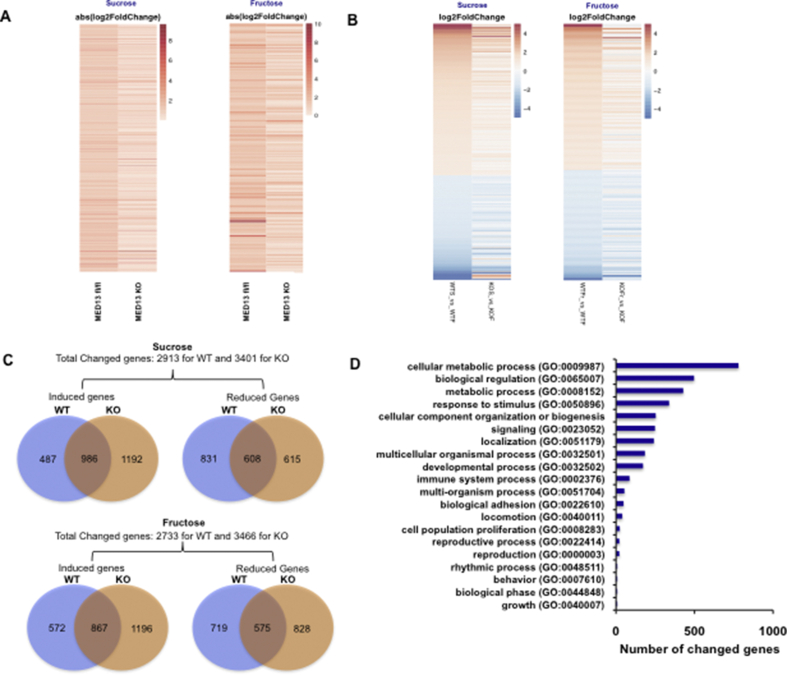


**Fig. s13:**
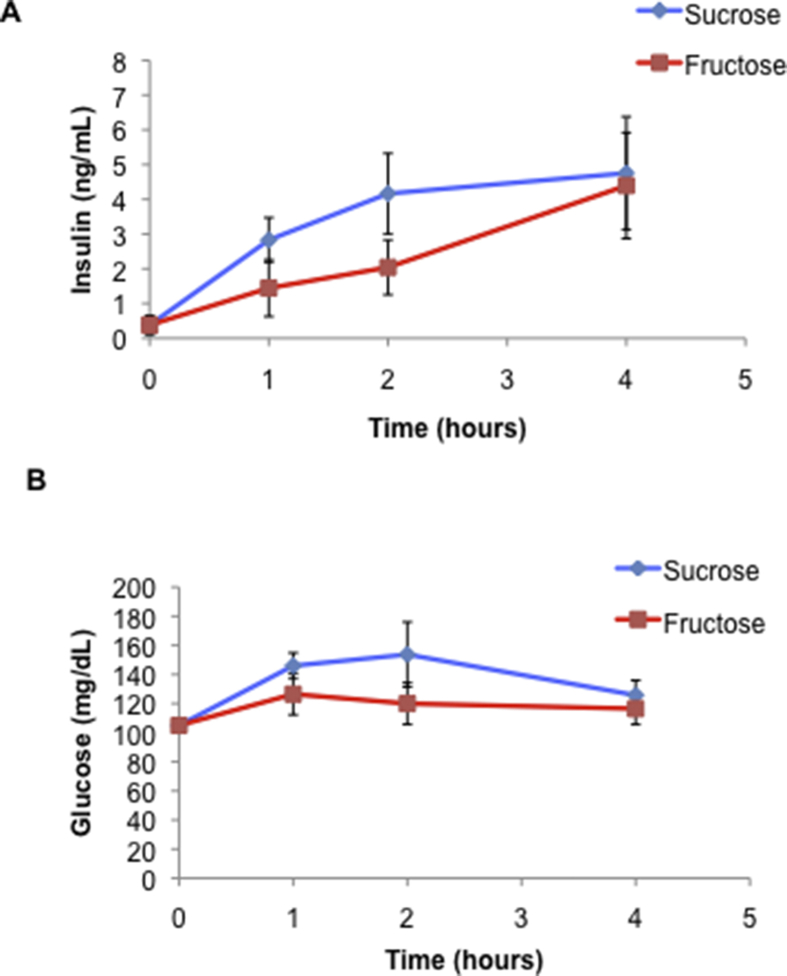


**Fig. s14:**
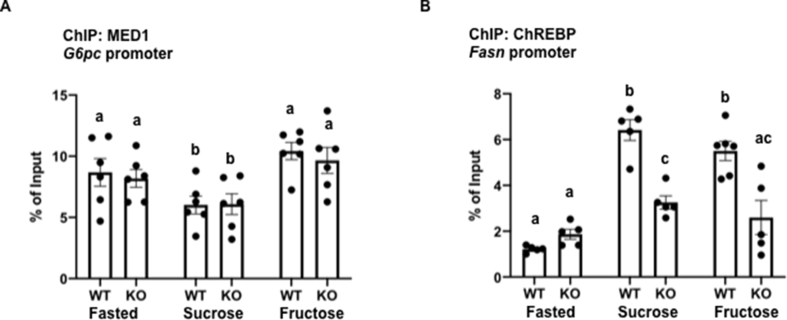


**Fig. s15:**
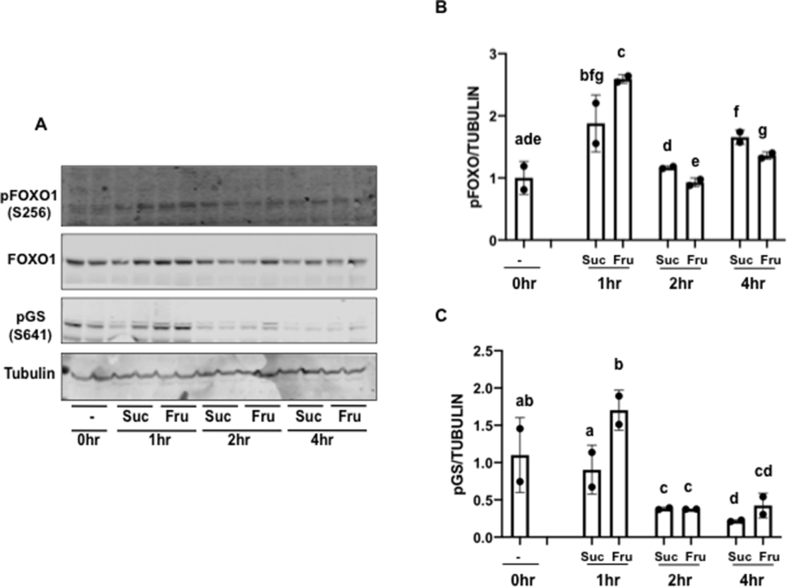


**Fig. s16:**
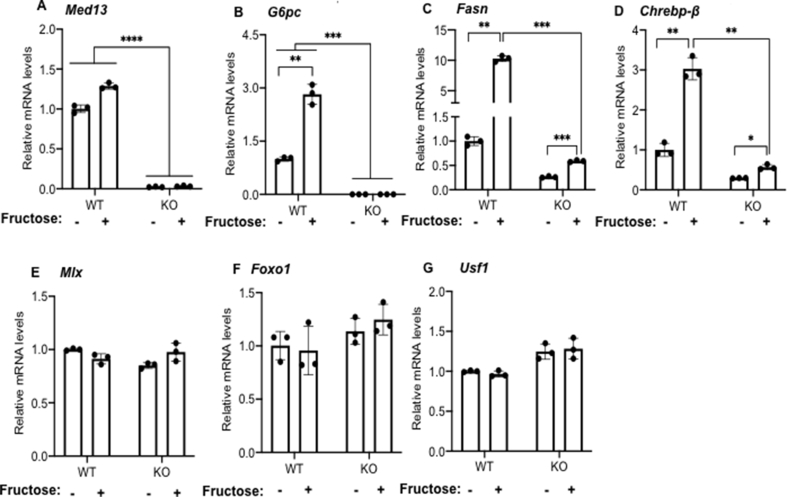


**Fig. s17:**
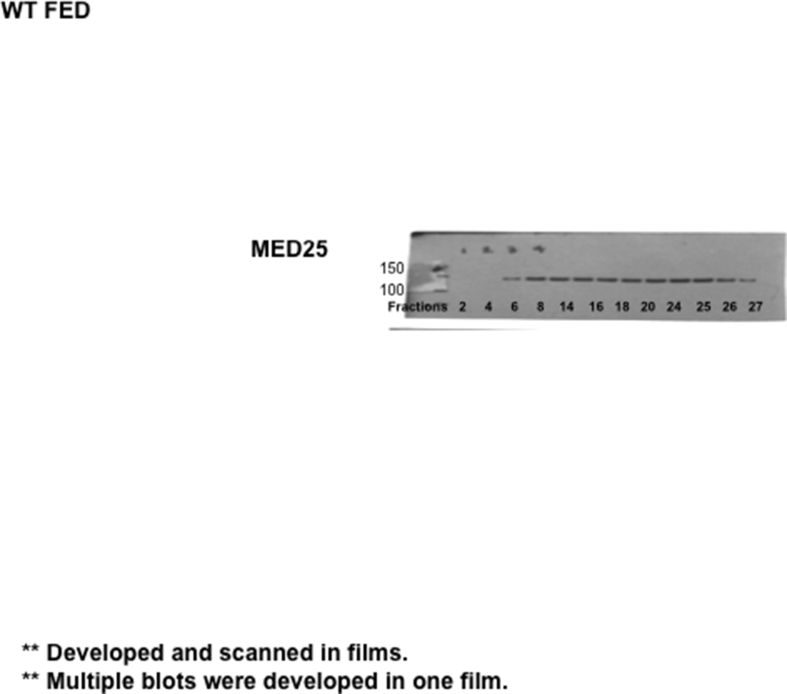


**Fig. s18:**
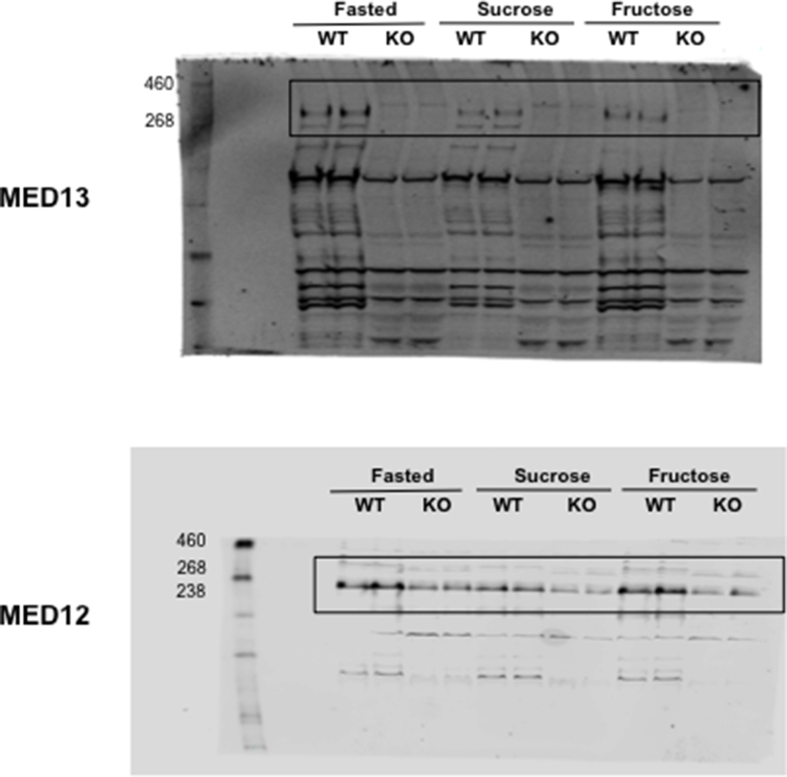


**Fig. s19:**
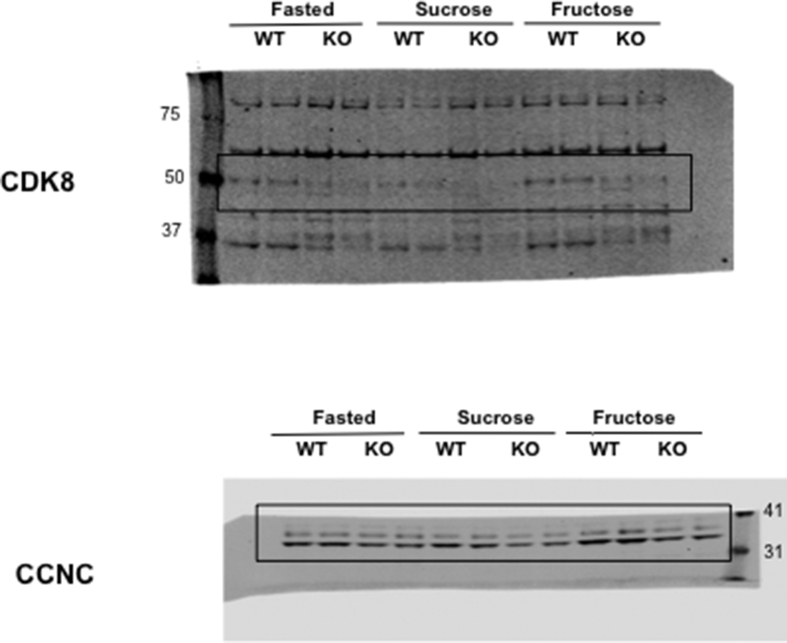


**Fig. s20:**
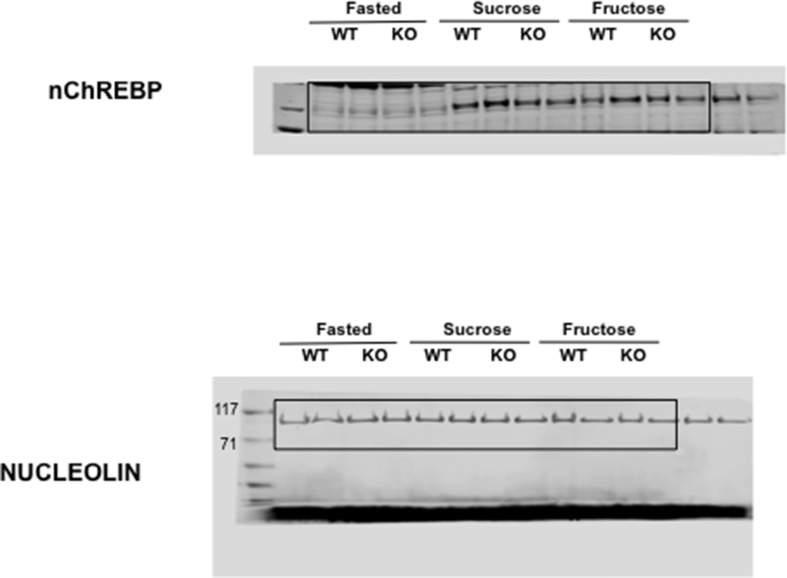


**Fig. s21:**
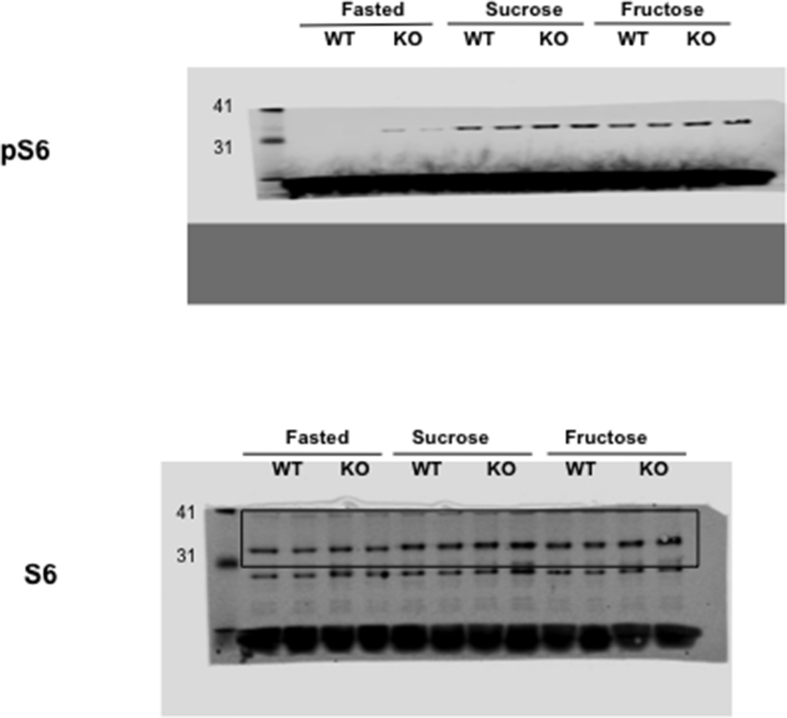


**Fig. s22:**
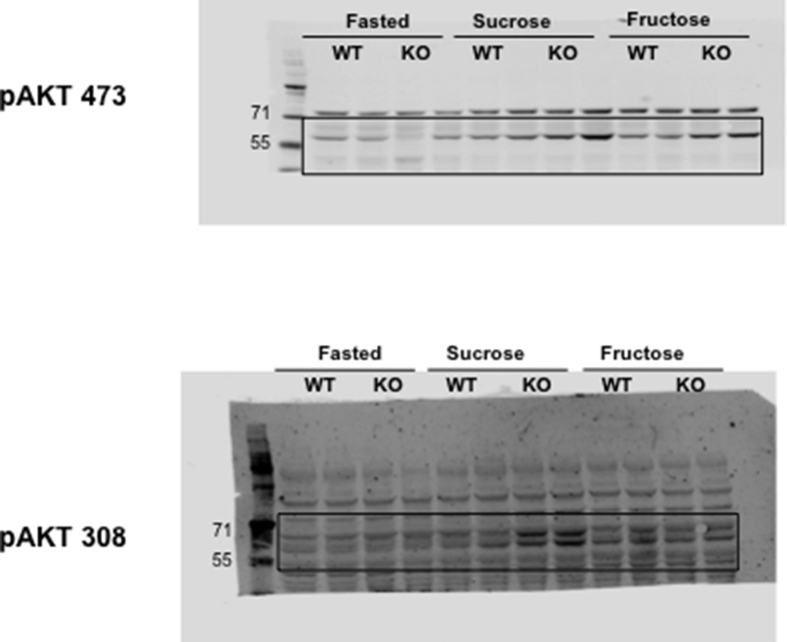


**Fig. s23:**
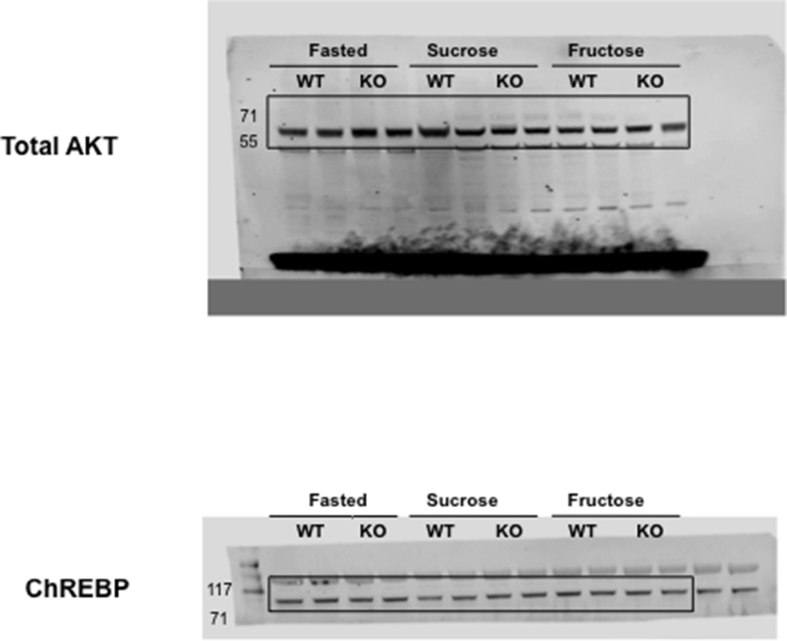


**Fig. s24:**
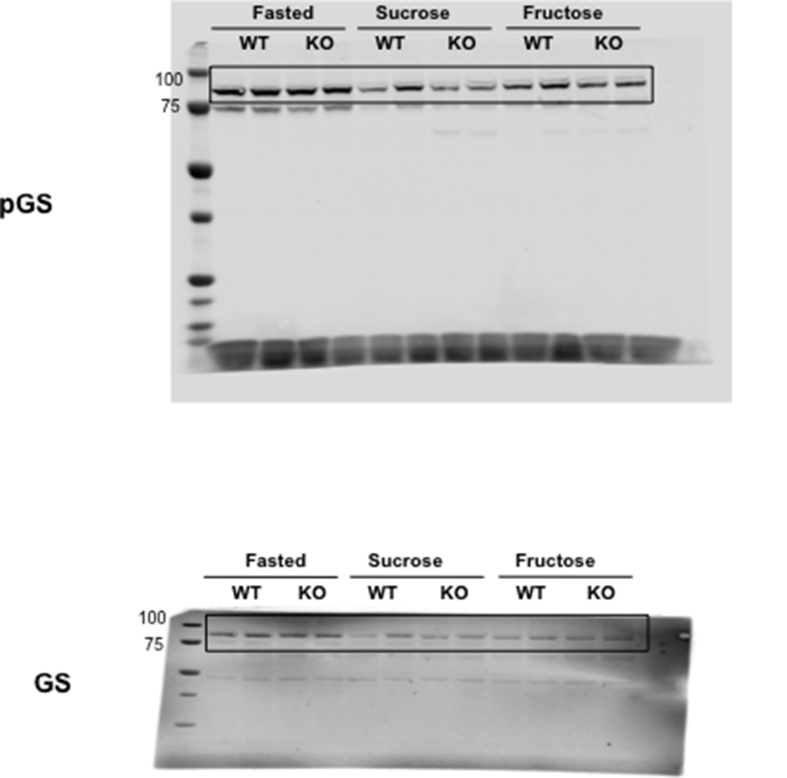


**Fig. s25:**
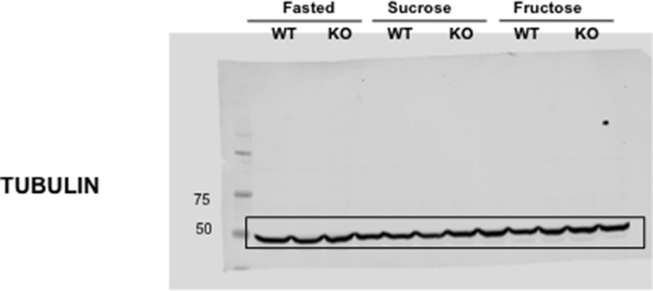


**Fig. s26:**
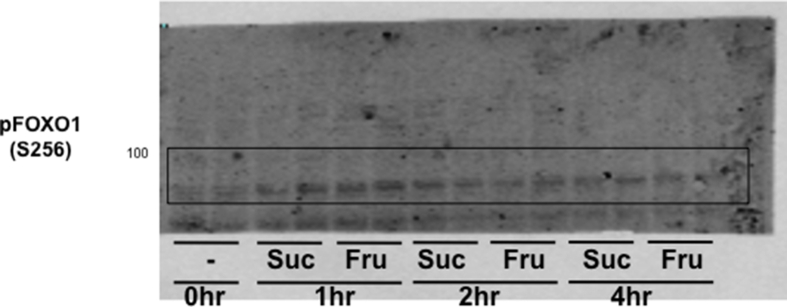


**Fig. s27:**
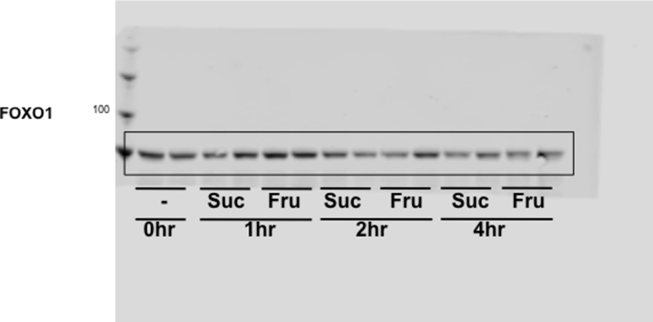


**Fig. s28:**
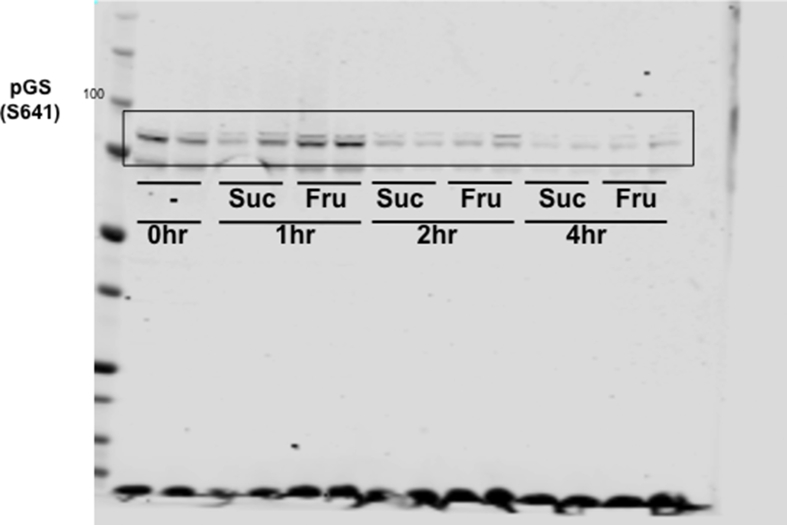


**Fig. s29:**
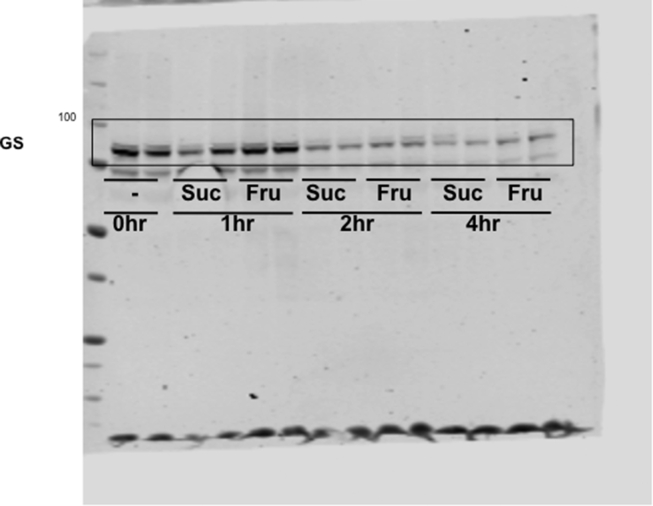


**Fig. s30:**
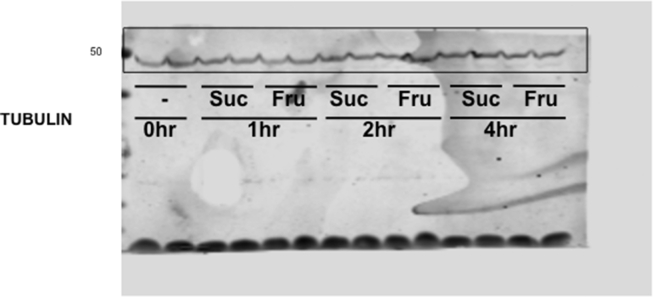


**Fig. s31:**
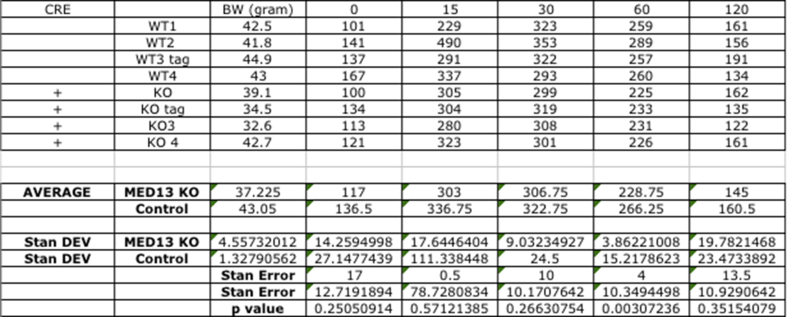


**Fig. s32:**
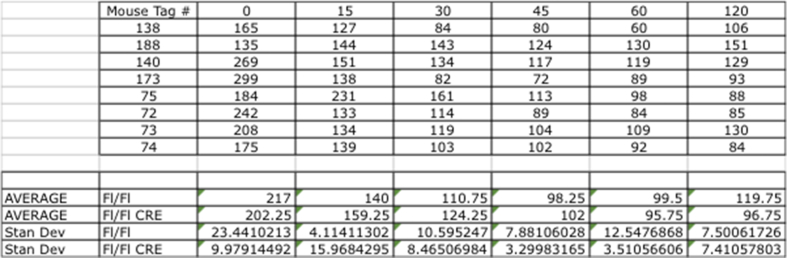


**Fig. s33:**
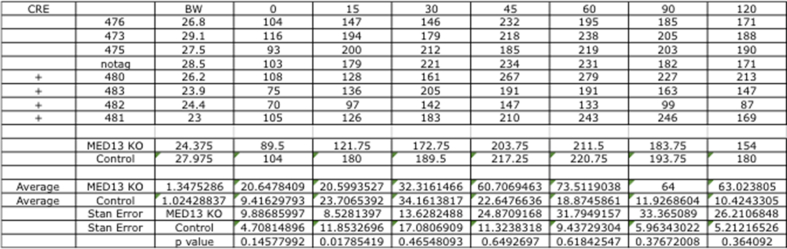


## Appendix A Supplementary data

Table S1 List of mouse oligonucleotide primer sequences for qRT-PCR. Table S1
