# Supplementary material for: The Mediator complex kinase module is necessary for fructose regulation of liver glycogen levels through induction of glucose-6-phosphatase catalytic subunit (G6pc)
Source: Mol Metab. 2021 Mar 31;48:101227. doi: 10.1016/j.molmet.2021.101227 (PMC8099662; doi:10.1016/j.molmet.2021.101227)
Supplement: Table S1 — List of mouse oligonucleotide primer sequences for qRT-PCR. [file mmc1.docx]

**SUPPLEMENTAL FIGURE LEGENDS**

**Table S1. List of mouse oligonucleotide primer sequences for qRT-PCR**

| **Primer** | **Sequence (forward)** | **Sequence (reverse)** |
| --- | --- | --- |
| *Fasn* | GGTCGTTTCTCCATTAAATTCTCAT | CTAGAAACTTTCCCAGAAATCTTCC |
| *Chrebp β* | TCTGCAGATCGCGTGGAG | CTTGTCCCGGCATAGCAAC |
| *Chrebp α* | ATCCGACACTCACCCACCTCTT | GCTCTCCAGATGGCGTTGTTCA |
| *Srebf1c* | GGAGCCATGGATTGCACATT | GGCCCGGGAAGTCACTGT |
| *Pck1* | TCTCTGATCCAGACCTTCCAA | GAAGTCCAGACCGTTATGCAG |
| *G6pc* | CTGTGAGACCGGACCAGGA | GACCATAACATAGTATACACCTGCTGC |
| *Mlx* | AGTCTTCAACGCCTCTATCTCTG | AATCTCTCGTAGAGTCTGTGGCTC |
| *Lxr α* | ATCGCCTTGCTGAAGACCTCTG | GATGGGGTTGATGAACTCCACC |
| *Usf1* | AAGTCAGAGGCTCCCAGGA | CGGCGCTCCACTTCGTTAT |
| *Med13* | CTTCACCGGAGCCAGAATAAA | GGAGACCACTTCCAACATCAA |
| *Gbe* | GGTCCAAGCTGAAGGTAGTTAT | GCCCAGTGTATCCAATCATAGT |
| *Phkb* | GCTCACTGTTACCCAGAGAATC | GCTTCATCATCCAAGGCAAAC |
| *Gs* | ACCAAGGCCAAAACGACAG | GGGCTCACATTGTTCTACTTGA |
| *Ppib* | CAAGCATGTGGTTTTCGGCA | GCTGTCTGTCTTGGTGCTCT |
|  |  |  |
| ***ChIP primer*** | **Sequence (forward)** | **Sequence (reverse)** |
| *G6pc promoter* | AGGTACCAAGGGAGGAAGGA | TGGAACCAGATGGGAAAGAG |
| *G6pc promoter 2* | CTCTGTCAAGCAGTGTGCCCAAGTTAATA | GTCTGTAGGTCAATCCAGCCCTGATCTT |
| *Fasn promoter* | TGTTCCCTATCCTGCCTACT | ATCGTGGCCCAGCTTTC |
| *Fasn promoter 2* | CCAGCTTATGGGCAGAATACA | AGCTTCTCCAACTTGGCTATC |
